# Digital Health Interventions to Promote Physical Activity Among Adolescents: Systematic Review

**DOI:** 10.2196/82395

**Published:** 2026-02-27

**Authors:** Rui Shi Fan, Jia Jun Jiang, Qing Yuan Zhou, Xin Yue Zhang, Zhou Hang Wu, Liu Ji

**Affiliations:** 1 College of Physical Education and Health East China Normal University ShangHai, Shanghai China; 2 Key Laboratory of Adolescent Health Assessment and Exercise Intervention of Ministry of Education, East China Normal University ShangHai China

**Keywords:** adolescents, physical activity, digital health interventions, systematic review, health promotion, behavior change, youth health promotion

## Abstract

**Background:**

Insufficient physical activity among adolescents is a major global public health concern. Digital health interventions (DHIs) have gained increasing attention as a promising approach to promoting physical activity in adolescents. However, existing systematic reviews predominantly focus on single-intervention formats or specific study designs, while reviews that integrate multiple DHIs and diverse study designs remain scarce.

**Objective:**

This systematic review aims to synthesize evidence from diverse DHIs and multiple study designs to assess their effectiveness in promoting physical activity among adolescents.

**Methods:**

The review protocol was registered in PROSPERO (International Prospective Register of Systematic Reviews; CRD420251117923). This systematic review searched literature published between January 1, 2014, and June 30, 2025, across Web of Science, PubMed, EBSCO, Scopus, Embase, the Cochrane Library, ProQuest, and Google Scholar. The final search was completed on August 3, 2025. Using the PICOS (population, intervention, comparator, outcomes, and study design) framework, the review included adolescents aged 10-19 years and focused on evidence-based research promoting physical activity through DHIs. The review was limited to peer-reviewed English-language literature and excluded studies solely focused on measurement tools, those not evaluating intervention effectiveness, or those not involving adolescents. Two reviewers independently screened studies and extracted data. Research quality was assessed using the Joanna Briggs Institute tool. Findings were synthesized through narrative synthesis and qualitative content analysis.

**Results:**

A total of 24 studies were included, involving approximately 12,183 adolescents. Study designs comprised 10 randomized controlled trials, 4 quasi-experimental studies, 3 quantitative research studies, 3 cross-sectional studies, and 4 mixed methods studies. Overall, 7 (29%) studies were of high quality, 16 (67%) were of moderate quality, and 1 (4%) was of low quality. Study populations included general adolescents as well as subgroups with specific health risks: insufficient physical activity (1/24, 4%), obesity or overweight (4/24, 17%), attention-deficit/hyperactivity disorder (1/24, 4%), cancer survivors (1/24, 4%), and at-risk youth (1/24, 4%). DHIs were categorized into 3 types: single-driver interventions (14/24, 58%), multimodal integrated interventions (7/24, 29%), and interaction-enhanced interventions (3/24, 13%). Most studies reported positive outcomes, including direct effectiveness (15/24, 63%), indirect effectiveness (8/24, 33%), and unclear effectiveness (1/24, 4%).

**Conclusions:**

This systematic review synthesizes evidence from diverse research designs and multiple types of DHIs, offering a more comprehensive perspective than previous reviews focused on single designs or technological formats. The results indicate that DHIs generally enhance adolescent physical activity levels, although their effectiveness varies considerably across intervention types and study designs. The review fills key research gaps and highlights the critical role of intervention adaptability and implementation context. It also addresses practical concerns, including adolescents with special health conditions, digital health inequalities, and technology dependency. Despite limitations related to methodological quality and insufficient follow-up, this review provides important evidence to inform practical application, policy development, and the equitable promotion of DHIs to enhance adolescent physical activity. Against the backdrop of rising global adolescent physical inactivity and widening health disparities, it also outlines directions for future high-quality research.

**Trial Registration:**

PROSPERO CRD420251117923; https://www.crd.york.ac.uk/PROSPERO/view/CRD420251117923

## Introduction

### Background

Adolescence represents a pivotal developmental period for the establishment of long-term healthy lifestyle behaviors. Engaging in moderate physical activity during this stage contributes to physical development, improves cardiorespiratory fitness, supports mental health regulation, and shapes future health trajectories, including the prevention and management of chronic diseases. However, the prevalence of insufficient physical activity among adolescents has increased markedly in recent years and is now widely recognized as a significant global public health concern affecting youth populations [1].

Against this backdrop, digital health interventions (DHIs), an emerging approach to health promotion, have been increasingly implemented among adolescent populations [2]. These interventions typically utilize digital technologies—such as mobile health (mHealth) apps, wearable devices, and online fitness platforms—to facilitate behavioral change among adolescents. They offer advantages such as high accessibility and real-time feedback, rendering them particularly suitable for this demographic group [3,4]. For example, within the domain of gamified mobile apps, fitness platforms such as “Nike Training Club” enhance daily exercise participation through mechanisms including instructional video tutorials and task-based check-ins. Wearable devices, such as Fitbit and Garmin, deliver real-time feedback on physical activity indicators, including step count, heart rate, and energy expenditure, thereby supporting users in enhancing self-regulation of physical activity [5]. Overall, DHIs exhibit growing diversity and adaptability in promoting physical activity among adolescents, while simultaneously presenting novel research opportunities and methodological challenges within the field.

As DHIs evolve, understanding the underlying behavioral mechanisms is key to optimizing intervention design and improving effectiveness. The Behavior Change Wheel (BCW) explains how DHIs influence behavior change and provides a foundation for developing targeted strategies. Research suggests that the COM-B (capability, opportunity, motivation—behavior) system is a robust framework for designing DHIs. The COM-B system elucidates the mechanisms underlying behavior change [6], explaining how different technologies and strategies operate across various contexts. Proposed by Michie et al [6], this model is central to the BCW and has been widely applied in health behavior research. It explains how multimechanism strategies work together to promote health behavior change. The system proposes that behavior requires 3 elements: (1) capability, (2) opportunity, and (3) motivation. These elements interact to form a behavioral system; for example, enhanced capability can increase motivation, favorable opportunities can facilitate the use of capability, and higher motivation can drive further pursuit and development.

In recent years, DHIs have undergone continuous innovation in content strategy. The content of such interventions has evolved from single-dimensional information delivery to multidimensional models that integrate behavioral incentives, motivational guidance, and social support. Similarly, intervention modalities have transitioned from single-medium approaches to integrated, multicomponent systems that combine mobile apps, wearable technologies, and social networking platforms. Despite these advancements, research on DHIs aimed at promoting physical activity among adolescents remains substantially underdeveloped. At the level of primary research, existing studies targeting physical activity promotion among children and adolescents demonstrate considerable diversity in intervention modalities; however, a standardized classification framework remains absent. Moreover, substantial variation is observed across studies with respect to intervention tools, implementation frequency, and intervention duration, contributing to pronounced heterogeneity in reported outcomes [7]. Simultaneously, methodological weaknesses persist in some studies—such as the absence of control groups, small sample sizes, short intervention periods, or a lack of long-term follow-up—thereby undermining the interpretability and external validity of the findings [8].

Existing reviews primarily focus on specific DHIs, with few systematic reviews encompassing multiple intervention types and diverse study designs. Previous reviews, such as that by Wang et al [9], focused on mHealth app interventions using randomized controlled trials (RCTs) and reported benefits for total physical activity, sedentary behavior, BMI, agility, and muscle strength in children and adolescents. He et al [10] examined smartphone-based interventions using RCTs, showing increases in total physical activity and step counts among children and adolescents, with app-based interventions proving more effective. Sequí-Domínguez et al [11] examined eHealth interventions in RCTs and found that they reduced sedentary time in children and adolescents.

The scope of these previous systematic reviews was relatively narrow, focusing solely on specific DHIs or intervention types. The included studies were limited to a single study design, resulting in incomplete interpretation of the evidence. Consequently, there remains a lack of comprehensive integration and comparison across multiple DHIs, as well as a paucity of reviews and further analyses encompassing diverse study designs.

### Objectives

Against this backdrop, this systematic review aims to conduct a focused analysis of the existing literature targeting adolescent populations. It seeks to identify and examine the capabilities of different research designs and various types of DHIs in promoting physical activity among adolescents, thereby addressing current evidence gaps. The review is intended to provide theoretical support and an empirical foundation for optimizing subsequent intervention design and research.

## Methods

### Review Design and Protocol Registration

This systematic review adopts a systematic methodology to comprehensively examine the current landscape, intervention types, and implementation effectiveness of DHIs aimed at promoting physical activity among adolescents in recent years. In addition, it evaluates and synthesizes the methodological quality and practical outcomes reported in the existing literature. The review strictly adheres to the PRISMA (Preferred Reporting Items for Systematic Reviews and Meta-Analyses) 2020 reporting guidelines [12], encompassing key steps including literature retrieval, screening, data extraction, and quality appraisal. To ensure scientific rigor and interstudy comparability, a structured risk-of-bias assessment was performed using the Joanna Briggs Institute (JBI) Critical Appraisal Tool [13]. The review protocol was prospectively registered with the international systematic review registry PROSPERO (International Prospective Register of Systematic Reviews; registration number CRD420251117923), and the study was conducted and reported in strict accordance with the preestablished protocol. The study protocol can be accessed via the corresponding registration record on the PROSPERO platform.

### Eligibility Criteria

#### Inclusion Criteria

The systematic review utilized the PICOS (population, intervention, comparator, outcomes, and study design) framework to define the following inclusion criteria.

- Population: Studies must include adolescent participants aged 10-19 years, with a primary focus on physical activity behaviors during this developmental stage.
- Intervention: Studies must examine DHIs aimed at promoting physical activity among adolescents. Eligible intervention formats include, but are not limited to, mHealth, wearable technologies, eHealth platforms, fitness tracking devices, virtual fitness programs, and gamified health apps.
- Comparison: Acceptable control conditions may include no intervention, traditional physical activity programs, or other nondigital health promotion approaches.
- Outcomes: Included studies must report outcomes related to adolescent physical activity behaviors, including but not limited to physical activity levels, exercise frequency, intensity of participation, sports involvement, and engagement in fitness-related activities.
- Study design: Eligible study designs include RCTs, quasi-experimental studies (QESs), quantitative research (QR), cross-sectional studies (CSSs), and mixed methods studies (MMSs).
- All included studies must be peer-reviewed publications written in English and published no later than June 30, 2025.

#### Exclusion Criteria

- Systematic reviews that exclusively examined physical activity assessment tools or indicators without evaluating intervention effectiveness.
- Systematic reviews involving participants who were not adolescents or whose data did not allow clear differentiation of adolescent subgroups.
- Systematic reviews classified as dissertations (master’s or doctoral), review articles, conference papers, or other non–peer-reviewed publications.
- Systematic reviews for which the full text was not accessible.
- Systematic reviews with unclear research designs or markedly low methodological quality.

### Information Sources

This systematic review retrieved relevant studies from multiple databases and information sources. Primary databases included PubMed (National Library of Medicine), Web of Science (Clarivate Analytics), EBSCO (EBSCOhost), Scopus (Elsevier), Embase (Elsevier), the Cochrane Library (Wiley Online Library), and ProQuest (ProQuest Platform), with Google Scholar serving as a supplementary search source. All databases were searched independently on their respective platforms, with no simultaneous searches conducted on a single platform.

To identify potentially overlooked studies, the research team conducted manual supplementary searches. This process involved reviewing the reference lists of included studies and examining references from relevant systematic reviews and meta-analyses. No additional studies or data were obtained from authors, experts, or other individuals. The review comprised 2 rounds of searching: the initial search was completed on June 10, 2025, for primary study identification, and a second, updated search was completed on August 3, 2025, to capture newly published studies. Both searches employed consistent search strategies.

### Search Strategy

The literature search strategy was constructed in accordance with the PRISMA-S (Preferred Reporting Items for Systematic Reviews and Meta-Analyses—Search Extension) guidelines to ensure transparency and reproducibility [14]. The search strategy was organized around 3 core concept categories. First, physical activity behaviors: “physical activity,” “exercise,” “physical fitness,” “sports participation,” and “fitness activity.” Second, DHIs: “digital health,” “mobile health,” “wearable device,” “electronic health,” and “fitness tracking device.” Third, study participants: “adolescent” and “teenager.” Search terms within the same concept category were combined using the Boolean operator “OR,” while different concepts were linked with “AND.” Search fields and expressions were adjusted according to database-specific syntax and subject heading systems (eg, Medical Subject Headings [MeSH]).

The search time frame spanned from January 1, 2014, to June 30, 2025, and was limited to English-language original empirical research. This systematic review did not utilize published standardized search filters; the retrieval strategy was specifically designed for this study and was not adapted or reused from previous systematic reviews or meta-analyses. The complete search terms and database-specific parameters are detailed in Multimedia Appendix 1. Beyond these methods, this systematic review did not search study registries, conduct purposeful browsing of conference proceedings, government websites, or other online resources; employ additional complementary search methods; or undergo a dedicated peer review of the search strategy.

### Selection Process

All search results were first imported into the EndNote reference management software (Clarivate Plc) for preliminary deduplication. Subsequently, the Rayyan online reference management platform was used for literature screening. The screening process was conducted independently by 2 researchers (RSF and JJJ) and comprised 2 stages: title and abstract screening, followed by full-text screening. Disagreements during screening were resolved through discussion and negotiation between the 2 researchers. If consensus could not be reached, a third researcher (XYZ) intervened to make the final decision, ensuring the objectivity and consistency of the screening process.

### Data Collection Process

After study inclusion, data were collected using preestablished standardized data extraction forms. Two researchers (RSF and JJJ) independently extracted data on study characteristics, including basic study information, methodological features, forms of DHIs, and intervention outcomes. When critical information was missing or unclear, the researchers contacted the corresponding author to obtain supplementary data. Discrepancies identified during data extraction were resolved through discussion and consensus, and unresolved disagreements were referred to a third researcher (XYZ) for deliberation and final adjudication.

### Study Outcomes (Data Items)

Data extraction was performed independently by 2 researchers using predefined standardized data extraction forms. Extracted data included primary and secondary outcomes. Primary outcomes comprised basic study information (eg, authors, study population characteristics, sample size), study design and methodological features, and specific forms of DHIs along with their effects on adolescent physical activity–related outcomes, summarized in conjunction with the reported outcome measures and overall intervention results.

Secondary outcomes included supplementary information supporting outcome classification and interpretation, such as geographic region, intervention duration, and follow-up or sample attrition rates. Disagreements during the process were resolved by a third researcher. Complete data extraction forms and the classification framework are presented in Tables 1 and 2.

**Table 1:** Overview of included studies (N=24).

Study	Age, mean (SD); grade; gender; condition; population	Region	Study design	Intervention duration	Digital health interventions	Effects of digital health interventions
Soltero et al [15]	14.9 (0.91); 53% female; obese/overweight	Spain	Quantitative research	12 weeks	Wearable device (Fitbit Watch) + SMS text messaging	High engagement in self-monitoring behavior and perceived increase in activity
Goodyear et al [16]	13-14; 53% female; general adolescents	The United Kingdom	Quantitative research	8 weeks	Wearable device (Fitbit) + virtual platform (with activity goal setting)	Encouraged youth to engage in more physical exercise
Van Dyck et al [17]	12-14; 49% female; general adolescents	Belgium	Quantitative research	1 week	SMS text messaging/chatbot + social media (Facebook) + SMS	Facebook and SMS text messaging were considered promising methods for physical activity interventions among vocational school adolescents
Dinç et al [18]	14.27 (0.44); 56% female; general adolescents	Turkey	Cross-sectional study	N/A^a^	mHealth^b^: smartphone app	Effectively improves physical activity levels and health awareness
Mojica et al [19]	11-14; 100% female; general adolescents	The United States	Cross-sectional study	N/A	Social media: cell phones, computers, game consoles, and internet	Increased physical activity and more frequent participation in daily physical education classes after the intervention
Ng et al [20]	11-15; 42% female; general adolescents	Finland	Cross-sectional study	N/A	Wearable device: physical activity tracker (smartwatches and heart rate monitors)	Positive correlation between physical activity behavior and use of wearables/apps; further research needed to verify relationship
Mendoza et al [21]	Intervention: 16.9 (1.5); 41.4% female; cancer survivors Control: 16.3 (SD 1.5); 40% female; cancer survivors	The United States	Randomized controlled trial	10 weeks	mHealth: Fitbit + Facebook	Demonstrates good feasibility and acceptability
Larsen et al [22]	14.7 (2.1); insufficient physical activity	The United States	Randomized controlled trial	12 weeks	Virtual platform: intervention website	Shows good feasibility and acceptability, with a significant increase in self-reported physical activity levels
Thompson et al [23]	14-17; 51.88% female; general adolescents (group: control, pedometer only, pedometer + goal prompts, and pedometer + goal prompts + self-determination theory texts)	The United States	Randomized controlled trial	12 weeks	mHealth: smartphone (SMS text messaging) + pedometer	Moderate increase in average daily steps and moderate-to-vigorous physical activity
Guthrie et al [24]	12-14; 55% female; general adolescents	The United States	Randomized controlled trial	6 weeks	Virtual platform: online intervention system (Zamzee)	Positive impact on moderate-to-vigorous physical activity levels
Direito et al [25]	14-17; general adolescents	New Zealand	Randomized controlled trial	8 weeks	mHealth: smartphone (immersive application)	Demonstrated feasibility
Chen et al [26]	14.9 (1.7); 42% female; obesity or overweight	The United States	Randomized controlled trial	6 months	mHealth: + wearable device + virtual platform: SMS text messaging, Fitbit Flex, and online educational program	Increased number of days per week engaged in physical activity
Caillaud et al [27]	Intervention: 10.9 (0.7); 51% female; general adolescents Control: 10.4 (0.5); 62% female; general adolescents	Australia	Randomized controlled trial	5 weeks	Virtual platform: app (iEngage Program)	Improved physical activity goals, academic performance, and moderate-to-vigorous physical activity levels
Egilsson et al [28]	15.6 (0.26); 41% female; general adolescents	Iceland	Randomized controlled trial	6 weeks	mHealth: smartphone (Mobile Health Program)	Feasible and usable
Staiano et al [29]	11.2 (0.8); 46% female; obesity or overweight	The United States	Randomized controlled trial	24 weeks	Game: Squad Intervention (gaming console)	Improved physical activity levels
Stasinaki et al [30]	PathMate2: 12.6 (range 11.4-16.9); 38.9% female; obesity or overweight Control: 13.7 (range 10.9-16.8); 46.2% female; obesity or overweight	Switzerland	Randomized controlled trial	5.5 months	mHealth: smartphone (PathMate2)	Significant and sustained improvements in physical capacity and body composition
Ortega and Cushing [31]	13-18; 45% female; general adolescents	The United States	Mixed methods study	27 days	SMS text messaging/chatbot: text bot (temporally augmented goal setting)	Increased participation, with observable changes in physical activity before and after the intervention
Willinger et al [32]	12.6 (1.7);27% female; general adolescents	Germany	Mixed methods study	4 months	mHealth: smartphone (the KIJANI app)	Helps oneself and others to be more active in daily life
Schoenfelder et al [33]	15.5 (1.4); 54% female; with attention-deficit/hyperactivity disorder	The United States	Mixed methods study	4 weeks	mHealth + Fitbit Flex + Facebook	Average weekly step count increased; intervention shows promise for promoting physical activity in adolescents with attention-deficit/hyperactivity disorder
Koorts et al [34]	13.7 (0.4); 49%female; general adolescents	Australia	Mixed methods study	12 weeks	mHealth + wearable device (wrist-worn Fitbit)	Perceived short-term positive effect on exercise motivation
Glaser et al [35]	Ninth to eleventh grade; 28% female; at-risk youth	Israel	Quasi-experimental study	8 months	Virtual platform: Friends Online Intervention Program—Zoom/video chat with online physical activities and dialogue	The program effectively increased physical activity among adolescents and reduced risky behaviors
Garde et al [36]	8-13; general adolescents	Canada	Quasi-experimental study	2 weeks	mHealth: smartphone game (Mobile Kids Monster Manor)	After the intervention, more physical activity was observed; the game’s role in promoting activity needs further validation
Mateo-Orcajada et al [37]	13.96 (1.21); 48% female; general adolescents	Spain	Quasi-experimental study	10 weeks	mHealth: mobile app	Postintervention improvements in physical activity level, body composition, and physical fitness quality
Cushing et al [38]	13-18; 75% female; general adolescents	The United States	Quasi-experimental study	3 weeks	SMS/chatbot: tailored SMS text messaging intervention	Increased physical activity and reduced sedentary behavior

^a^N/A: not applicable.

^b^mHealth: mobile health.

**Table 2:** Effectiveness of DHIs^a^ to promote PA^b^ in adolescents: a classification and ranking by intervention type.

Study			Measurement		Key findings		Result		Effect
**Single-driver intervention**									
	Dinç et al [18]	Questionnaire		PA-themed (40.5%) and healthy nutrition–themed (33.3%) apps were the most frequently used. Adolescents who preferred PA apps had significantly higher healthy lifestyle belief scores (mean 61.82, SD 0.70) than those who preferred Ministry of Health apps (mean 43.54, SD 2.91; P<.001). Similarly, users of self-monitoring health apps scored higher (mean 68.20, SD 0.28) than those using sleep-monitoring apps (mean 55.35, SD 1.24; P<.001).		Intrinsic motivation increased		I^c^	
	Mojica et al [19]	App data		Girls who owned a mobile phone were more likely than those who did not to engage in PA for more than 5 days in the past week (odds ratio 5.5, 95% CI 2.1-14) and to participate in daily physical education classes (odds ratio 2.6, 95% CI 1.1-5.9).		Enhanced PA		D^d^	
	Mendoza et al [21]	Measurable PA + questionnaire		Exploratory analyses found no significant differences in MVPAe (4.4 vs 5.0 minutes/day; P=.92) or sedentary time (–4.5 vs 1.0 minutes/day; P=.73) between the intervention and control groups. Mean differences were reported for these outcomes, with P values indicating no significant effects. Some modest differences were observed for select subscales of quality of life and motivation for PA.		Intervention feasible/acceptable		I	
	Larsen et al [22]	Questionnaire + semistructured interview + accelerometer measurement		According to physical activity recall measurements, participants’ MVPA time increased to an average of 79.4 (SD 46.8) minutes at follow-up, representing a per capita increase of 58.8 (SD 46.3) minutes. This difference was statistically significant (P<.001). Overall, PA levels showed a significant improvement from baseline to 12 weeks (P<.001).		Enhanced PA		D	
	Thompson et al [23]	Measurable PA + questionnaire		Program satisfaction: The mean score was 17.47/20, with no significant differences between groups (P=.068). The feasibility criterion—a program satisfaction score of ≥15 out of 20 points—was met. Internal consistency: All measures exceeded the feasibility criterion (α≥.70), with the lowest α being .74 for social desirability. PA trends: The all-intervention group showed the greatest increases in steps (+317.8 steps/day) and MVPA (+1.73 minutes/day) compared with other groups. However, no significant group-by-time interactions were observed (P>.05).		Enhanced PA		D	
	Guthrie et al [24]	Zamzee data		Participants receiving the Zamzee intervention exhibited average MVPA levels 54% higher than the passive control group (P<.0001) and 68% higher than the active control group using commercially available active video games (P<.0001).		Enhanced PA compared with the control group		D	
	Direito et al [25]	Self-report + accelerometer measurement		N/Af		The intervention will further validate its effectiveness.		I	
	Caillaud et al [27]	Accelerometer measurement + Physical Activity Questionnaire for Children + fitness test + iEngage app recording		Participants increased their daily step count by 30% (+2647 steps/day, P<.001), and the proportion of days exceeding 11,000 steps rose to 48%.		Enhanced PA		D	
	Egilsson et al [28]	Scale + app data		Self-reported daily PA increased by 19.61% in the intervention group but decreased by 26.21% in the control group. Self-efficacy levels increased by 8.23% in the intervention arm, compared with a 3.03% decrease in the control group.		Enhanced PA		D	
	Ortega and Cushing [31]	Accelerometer + questionnaire		The temporally augmented goal setting intervention was generally feasible, acceptable, and technically and functionally reliable. Adolescents demonstrated adequate levels of engagement. Changes in MVPA from pre- to postintervention were small, approximately a 2-minute increase.		Enhanced PA		D	
	Willinger et al [32]	Semistructured interviews + questionnaires		Most participants (n=16) believed that using KIJANI would increase their PA levels.		Enhanced PA		D	
	Garde et al [36]	PA monitor		During the intervention week, both groups showed significant increases in PA levels (1191 and 796 steps/day, respectively; P=.01). In the game group, children with higher BMI z scores exhibited greater PA levels, with an average increase of 964 steps/day for each unit increase in BMI z score (P=.03; 95% CI 98-1829).		Further validation is required.		U^g^	
	Mateo-Orcajada et al [37]	Questionnaire + physical fitness test + anthropometric measurements		Compared with the control group, adolescents in the experimental group demonstrated higher levels of PA, along with improvements in body composition and physical fitness following the intervention.		Enhanced PA compared with the control group and improvement in health indicators		D	
	Cushing et al [38]	Questionnaire + accelerometer		Compared with the attention control group, the intervention group spent an average of 20.84 minutes more per day in MVPA (β=20.84, SE 8.19).		Enhanced PA compared with the control group		D	
**Multimodal integrated intervention**									
	Soltero et al [15]	Interview		Young adults reported increases in both PA and self-efficacy for PA.		Intervention feasible and acceptable		I	
	Goodyear et al [16]	Interview		Daily goals of 10,000 steps and calorie burn motivate young people to engage in more PA.		Exercise goals encourage PA		I	
	Van Dyck et al [17]	Questionnaire + interview		Participants indicated that SMS text messages provide a simple way to receive information about PA, while Facebook group pages are ideal for sharing information with others.		The intervention shows promise as a method for increasing PA		I	
	Ng et al [20]	Scale		Compared with nonusers, app users had higher odds of engaging in daily MVPA (males: ORh 1.27, 95% CI 1.04-1.55; females: OR 1.49, 95% CI 1.20-1.85) and of being members of sports clubs (males: OR 1.37, 95% CI 1.15-1.62; females: OR 1.25, 95% CI 1.07-1.50). These associations were even stronger among wearable device users, with MVPA ORs up to 2.25. Male wearable users also had higher odds of engaging in active travel (OR 1.39, 95% CI 1.04-1.86).		Compared with the control group, the intervention showed a positive correlation with PA.		I	
	Chen et al [26]	Anthropometric measurements + questionnaire + Fitbit Flex data + interview		Compared with participants in the control group, those in the mobile phone–based intervention showed significant improvements in BMI (z=–4.37, P=.001), diastolic blood pressure (z=–3.23, P=.001), PA days/week (z=2.58, P=.01), TV and computer time (z=–3.34, P=.001), servings of fruits and vegetables per day (z=2.74, P=.006), servings of soda and sweetened drinks (z=–3.19, P=.001), PA self-efficacy (z=2.75, P=.006), and dietary self-efficacy (z=5.05, P=.001). Medium to large effect sizes were observed for these outcomes.		Enhanced PA and increased self-efficacy.		D	
	Schoenfelder et al [33]	Quantitative measures + Fitbit Flex data + questionnaires + interview		Participants significantly increased their average weekly step count and showed improvements in attention-deficit/hyperactivity disorder inattention symptoms.		Enhanced PA		D	
	Koorts et al [34]	Questionnaire + interview		Adolescents perceived the intervention content as easy to understand (100/120, 83.3%) and the Fitbit as easy to use (112/120, 93.3%). Half of the adolescents found the SMS text messages useful (61/120, 50.8%), while 47.5% (57/120) liked the weekly challenges, and 38.3% (46/120) liked the Facebook videos. Teenagers reported that the Fitbit Flex increased their awareness of PA and their likelihood of being more active in the short term.		Enhanced PA compared with the control group.		I	
**Interaction-enhanced intervention**									
	Staiano et al [29]	Anthropometry + full-body scanner + accelerometer + questionnaire		The intervention group showed a significant reduction in BMI z score, with a marginal difference in the intent-to-treat analysis (mean −0.06, SE 0.03 for the intervention group vs mean 0.02, SE 0.03 for the control group, P=.065). Compared with the control group, the intervention group also showed improvements in systolic blood pressure, diastolic blood pressure, total cholesterol, low-density lipoprotein-cholesterol, and MVPA (all P<.05).		Enhanced PA and improvement in health indicators		D	
	Stasinaki et al [30]	Physical fitness test (Dordel-Koch test) + questionnaire + application data		Both groups showed significant improvements in muscle mass, strength, and agility at T2, whereas only the PathMate2 group demonstrated a significant reduction in body fat at both T1 and T2. The average daily PathMate2 app usage rate was 71.5%.		Enhanced PA and improvement in health indicators		D	
	Glaser et al [35]	Questionnaire		Compared with the control group, the intervention group experienced a 57% increase in PA.		Enhanced PA		D	

^a^DHI: digital health intervention.

^b^PA: physical activity.

^c^DHIs are indirectly effective in promoting PA among adolescents (indirectly effective).

^d^DHIs are directly effective in promoting PA among adolescents (directly effective).

^e^MVPA: moderate-to-vigorous physical activity.

^f^N/A: not applicable.

^g^U: The effectiveness of DHIs in promoting PA among adolescents remains unclear (unclear).

^h^OR: odds ratio.

### Study Risk-of-Bias Assessment

To ensure methodological rigor and assess internal validity, structured risk-of-bias assessments were conducted using JBI critical appraisal tools, each aligned with the corresponding study design. This included assessment checklists applicable to CSSs, RCTs, QESs, QR, and MMSs [13].

**Figure 1:**
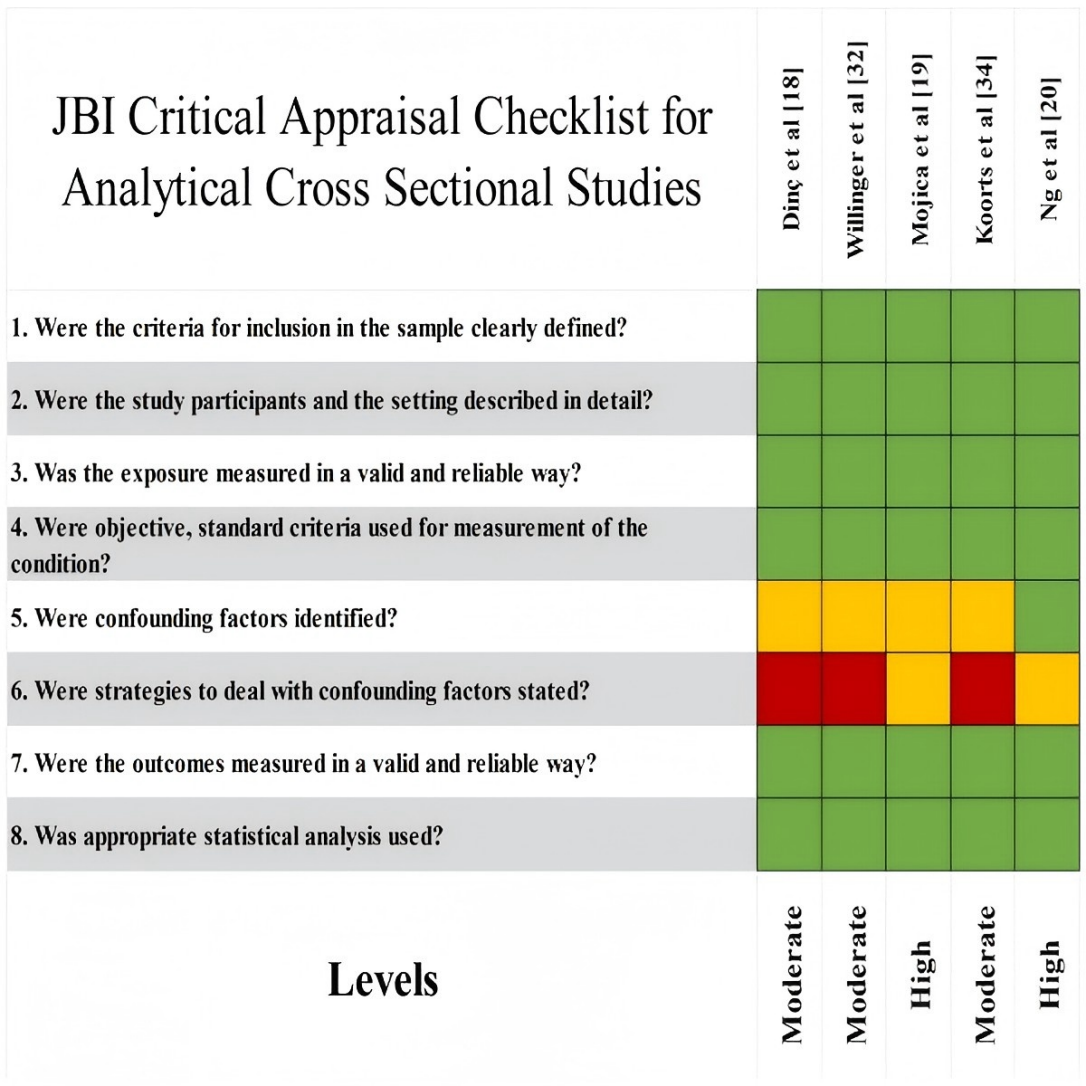
Quality assessment tool: cross-sectional studies. JBI: Joanna Briggs Institute.

**Figure 2:**
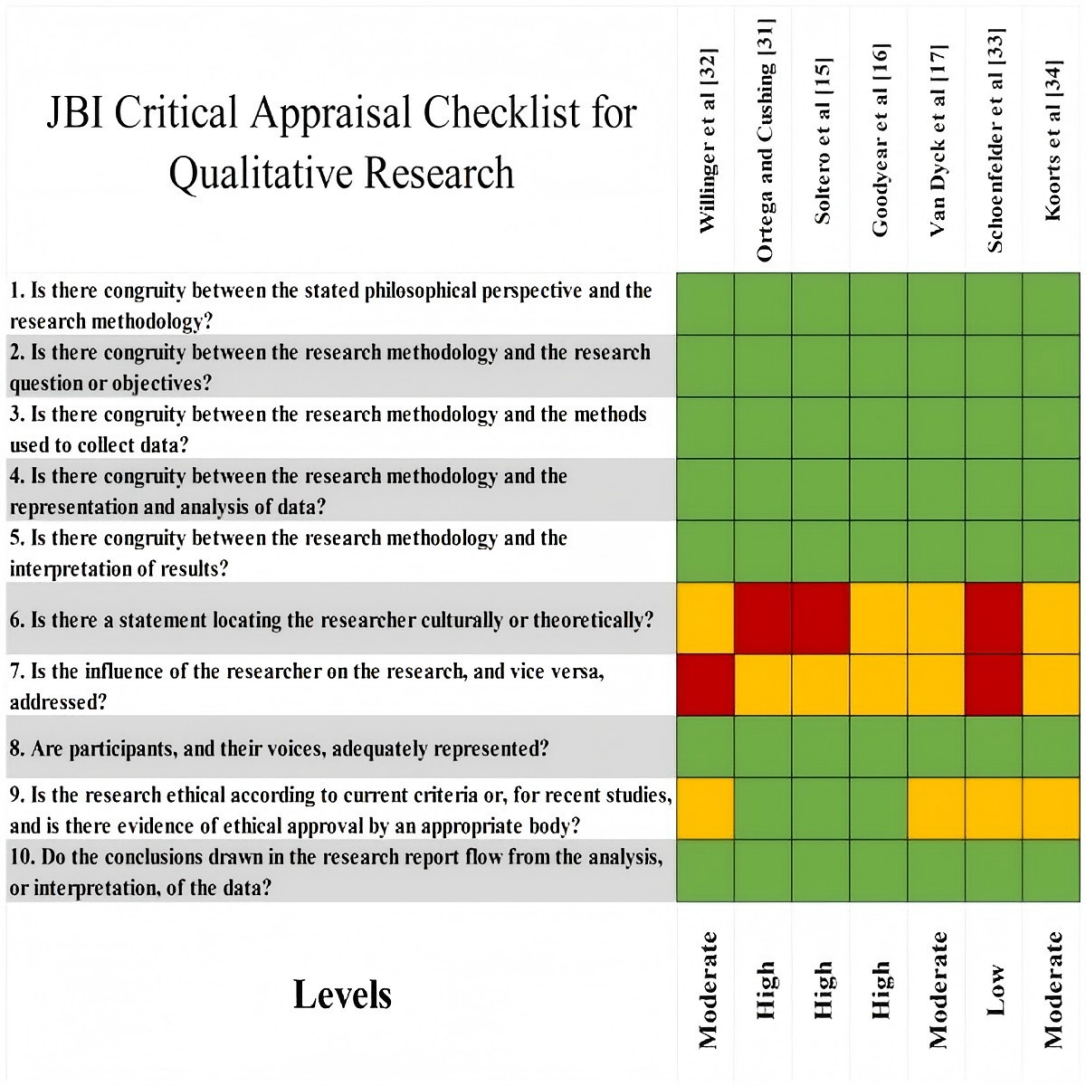
Quality assessment tool: qualitative research. JBI: Joanna Briggs Institute.

**Figure 3:**
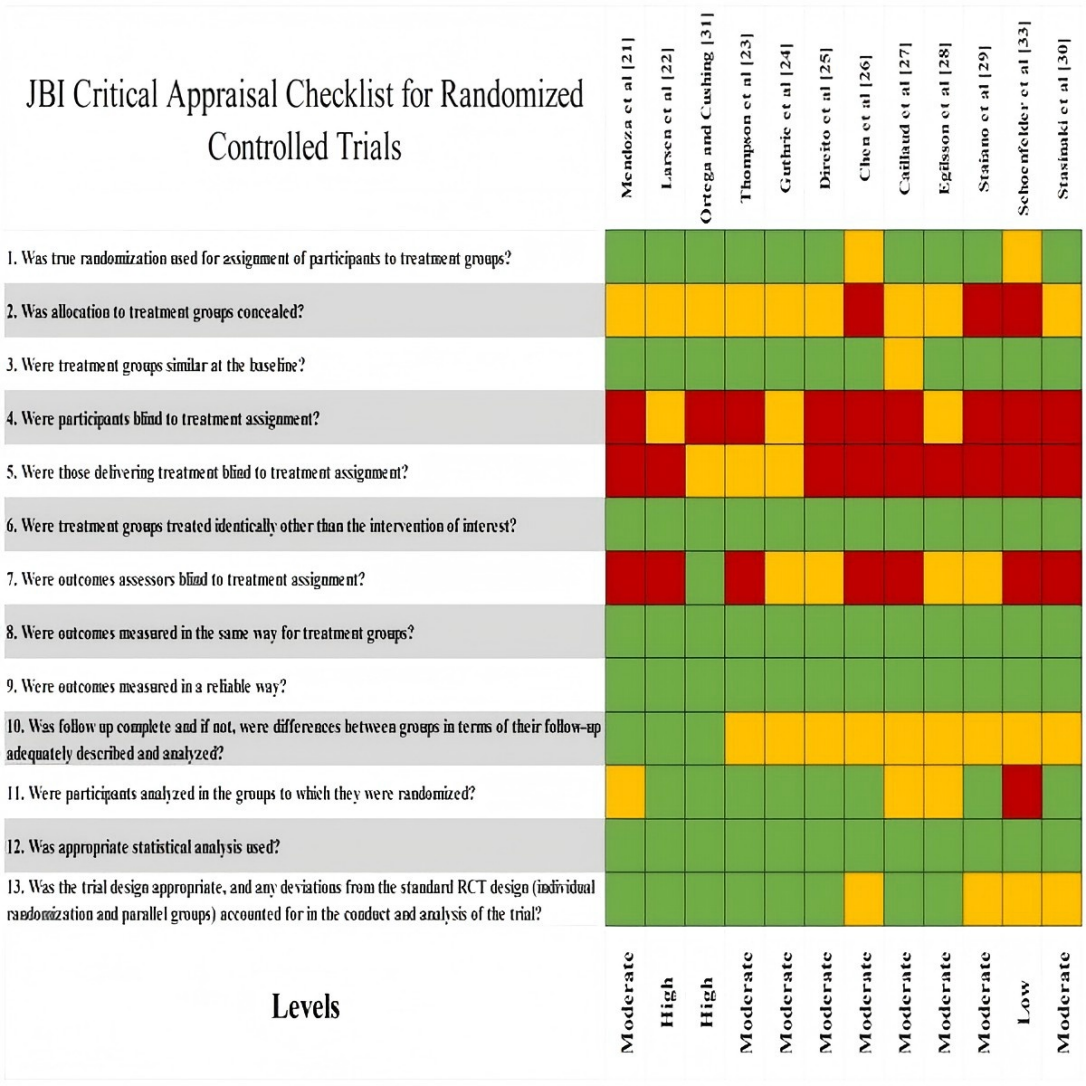
Quality assessment tool: randomized controlled trials. JBI: Joanna Briggs Institute.

**Figure 4:**
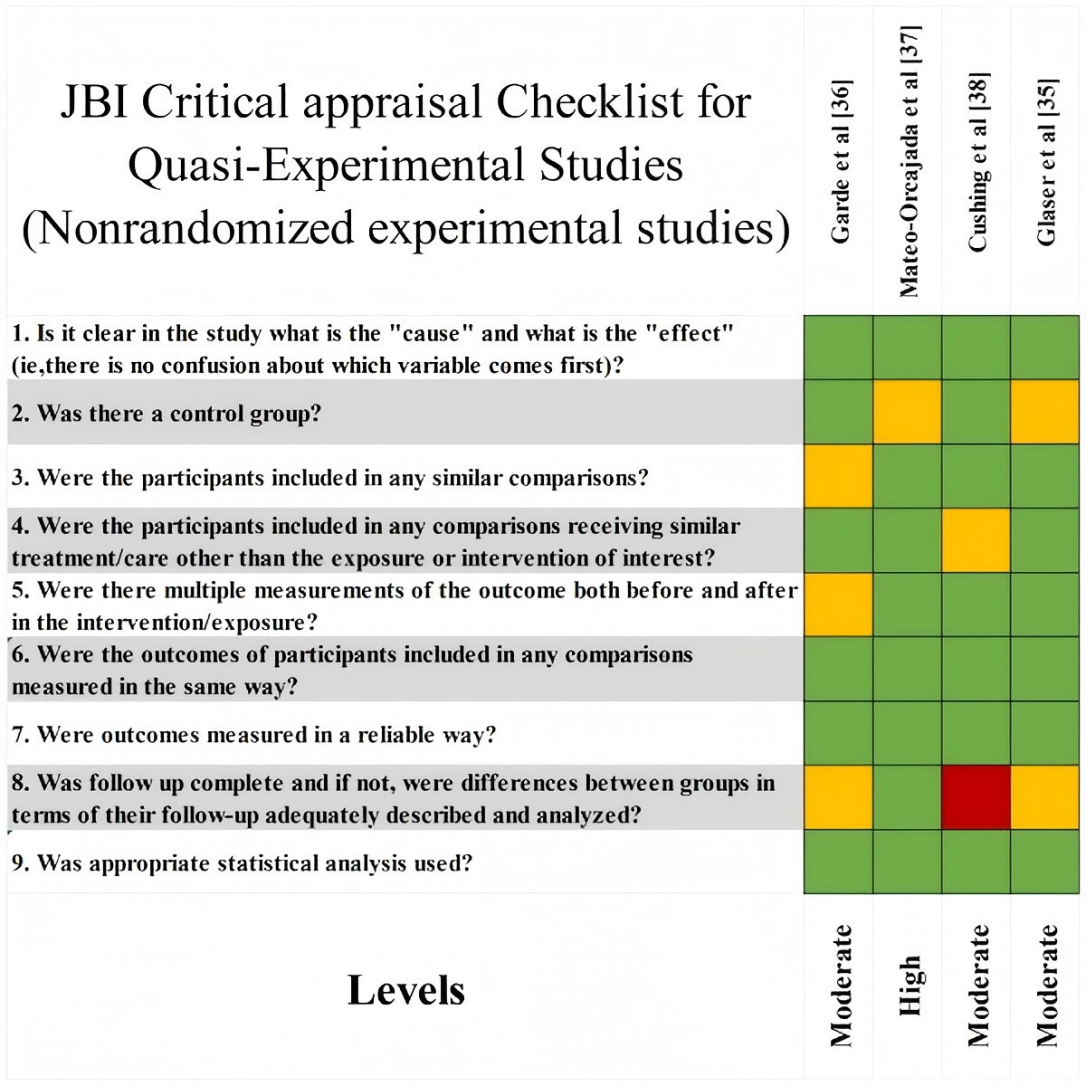
Quality assessment tool: quasi-experimental studies. JBI: Joanna Briggs Institute.

Risk of bias was assessed independently by 2 researchers using a 3-color coding system: green for “met,” yellow for “partially met,” and red for “not met.” Based on these assessments, each study’s methodological quality was categorized as high, moderate, or low. Disagreements were resolved through discussion and consensus, and if consensus was not reached, a third researcher adjudicated. Studies with reliability concerns—such as bias, unclear criteria, incomplete reporting, inappropriate methods, or insufficient justification—were flagged for further discussion and potential exclusion. No studies were excluded solely based on quality scores. Risk of bias was incorporated into the narrative synthesis to contextualize the strengths and limitations of the evidence. Detailed assessment results and scoring criteria are presented in Figures 1-4.

### Effect Measures

Effect measures reported in the studies were diverse, encompassing both quantitative metrics and qualitative outcomes. Quantitative studies primarily presented intervention effects through comparisons of mean values or change values (eg, physical activity duration, step count, and their changes before and after intervention or between intervention and control groups), odds ratios, and regression model estimates, supplemented by statistical indicators such as CIs and *P* values to support conclusions. Some studies reported only the direction of change or statistical significance of outcomes without providing uniformly convertible effect sizes.

Additionally, some studies reported intervention effects through qualitative descriptions (eg, user experience, satisfaction, or perceived changes). Given the inconsistencies in effect measure formats and statistical reporting methods, this review did not uniformly convert or pool effect sizes. Instead, it focused on comprehensively describing the directional trends and overall performance of intervention effects.

### Synthesis Methods

Given the substantial differences among included studies in research design, intervention modalities, outcome measurement, and statistical analysis methods, and considering that many studies did not report effect size parameters suitable for uniform conversion or pooling, this systematic review did not conduct a quantitative meta-analysis to avoid producing statistically unstable or potentially misleading pooled results. Prerequisites for meta-analysis include comparability or reasonable convertibility of outcome measurements and effect size definitions across studies. However, the included studies employed both self-reported and objectively measured outcomes, with inconsistent measurement time points, making these conditions unattainable.

The research team therefore employed a narrative synthesis and qualitative content analysis approach to integrate the findings. During synthesis, a classification framework based on core forms of DHIs and their effect characteristics was developed to determine study applicability across synthesis units and to facilitate group comparisons. This classification and synthesis were performed independently by 2 researchers, with disagreements resolved through discussion and, when necessary, adjudicated by a third researcher. Findings were primarily presented using structured tables and typological narratives (Tables 1 and 2), with interpretations of outcome variations grounded in intervention formats, study designs, and implementation characteristics. As no statistical synthesis was conducted, this review did not perform formal heterogeneity testing or sensitivity analyses.

### Reporting Bias Assessment

This systematic review employed a narrative synthesis and did not perform statistical tests for publication bias. Given the absence of a quantitative meta-analysis and the substantial heterogeneity in study design and outcome reporting across included studies, methods such as funnel plots were considered inapplicable. During evidence synthesis and result interpretation, the research team conducted a qualitative assessment of potential reporting bias. By comparing the consistency between study objectives, methods, and reported outcomes, and by incorporating study registration information (where available) and author explanations, the team cautiously discussed the potential impact of missing results on study conclusions.

### Certainty Assessment

This systematic review conducted a qualitative synthesis of the overall credibility of the evidence, considering the design types of included studies, the results of the JBI methodological quality assessment, and the consistency of study conclusions across different studies. Given that no quantitative meta-analysis was performed and the included studies exhibited substantial heterogeneity in design types and outcome measures, quantitative evidence grading tools such as GRADE (Grading of Recommendations, Assessment, Development and Evaluation) were not applied. The assessment of evidence certainty was conducted independently by 2 researchers (RSF and JJJ). The reliability and limitations of the evidence were interpreted in the “Results” and “Discussion” sections in conjunction with specific study findings.

### Ethical Approval

Ethics approval was not required for this systematic review.

## Results

### Study Selection

**Figure 5:**
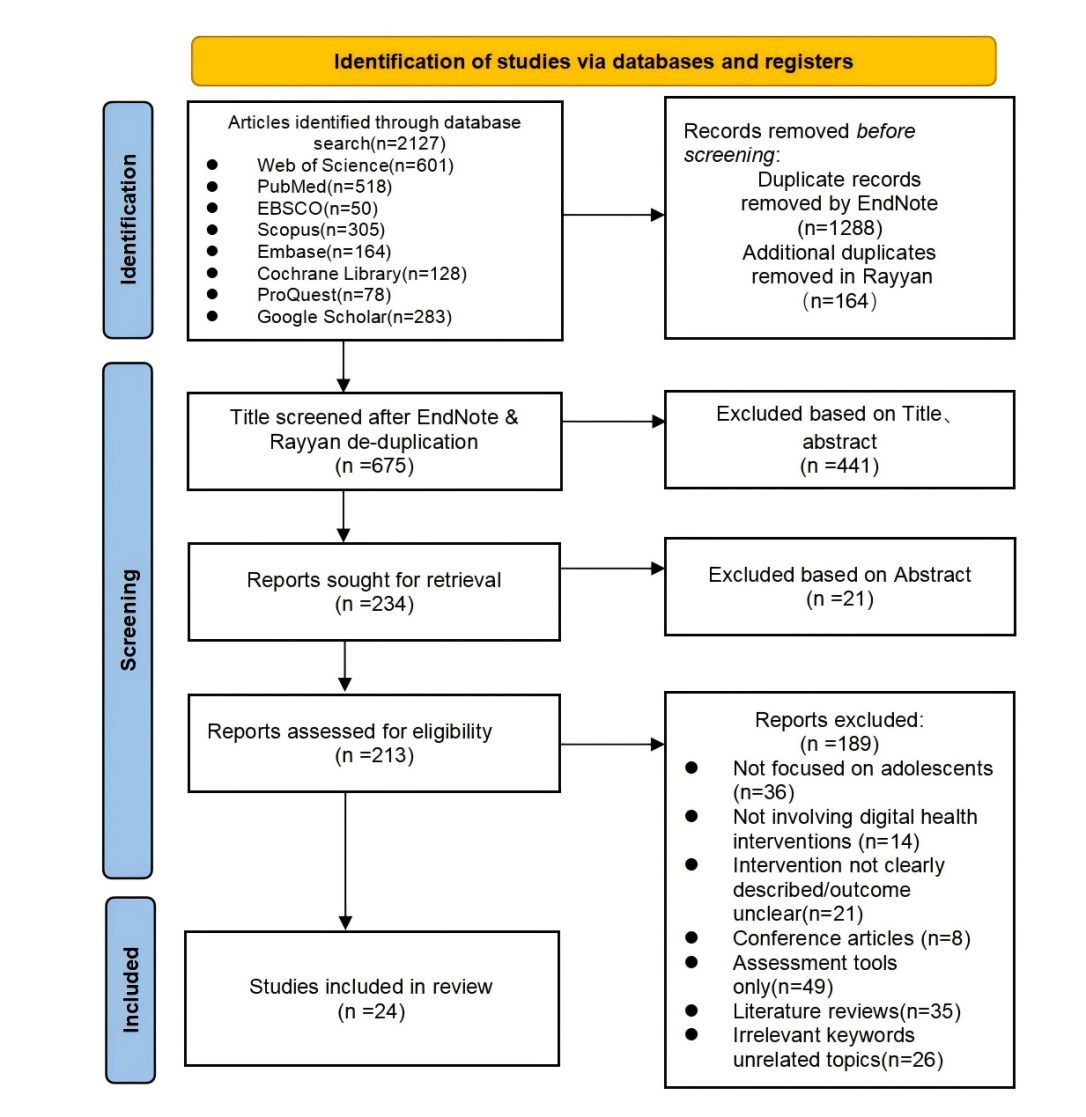
PRISMA Flow Diagram of the Study Selection Process.

A total of 2127 English-language articles were initially retrieved from 7 major databases, including Web of Science (n=601), PubMed (n=518), EBSCO (n=50), Scopus (n=305), Embase (n=164), Cochrane Library (n=128), ProQuest (n=78), and Google Scholar (n=283). After initial deduplication using EndNote, 1288 duplicate records were removed. Subsequently, an additional 164 duplicates were removed using the Rayyan online literature management platform, resulting in 675 records retained for title and abstract screening. Following this initial screening, 213 articles remained for full-text assessment. Of these, 189 articles were excluded based on predefined exclusion criteria, including study population, intervention characteristics, and research design. The specific reasons for exclusion were as follows: studies not focused on adolescents (n=36), absence of DHIs (n=14), insufficient clarity in intervention or outcome measures (n=21), conference abstracts (n=8), studies focused solely on instrument validation (n=49), review articles (n=35), and studies with unrelated topics (n=26). Ultimately, 24 English-language studies met the inclusion criteria and were included in the final qualitative synthesis of this systematic review (see Figure 5 and Multimedia Appendix 2).

### Study Characteristics

#### Overview

This systematic review included 24 peer-reviewed articles published between 2014 and 2025, spanning multiple countries and regions, thereby reflecting the global uptake of DHIs among adolescents. The included studies employed a range of methodological designs, including RCTs, MMS, QR, and other empirical approaches. All interventions targeted adolescents aged 10-19 years, including the general population, individuals with insufficient physical activity, and those with specific health conditions. Sample sizes ranged from 10 to 9940 participants, with the majority of studies involving fewer than 500 individuals. Intervention duration ranged from 2 weeks to 8 months. The included studies employed diverse intervention formats, utilizing either single-modality or multicomponent digital technology approaches. Detailed study characteristics are summarized in Table 1.

#### Participant Characteristics

This systematic review encompassed approximately 12,183 adolescent participants. Sample sizes varied widely, ranging from 10 to 9,940 participants. The overall mean sample size was approximately 507, whereas the median was substantially lower at 46. Most studies employed small-to-medium sample sizes, with a few large-scale studies exerting a disproportionate influence on the overall mean. Among studies that reported age data, participants’ age ranged from 8 to 19 years, with the highest concentration in the 13-15 age group, representing over one-third of the total sample. The study populations included both general adolescent cohorts and specific subgroups characterized by distinct health risks or behavioral profiles. Of the total 24 studies, 8 (33%) explicitly targeted specific subgroups, including 4 (17%) on adolescents with obesity or overweight, 1 (4%) on adolescents with insufficient physical activity, 1 (4%) on individuals with attention-deficit/hyperactivity disorder, 1 (4%) on cancer survivors, and 1 (4%) on at-risk adolescents. Additional details are provided in Multimedia Appendix 3.

#### Geographic Distribution

The 24 included studies were conducted across multiple countries and regions. North America accounted for the highest concentration, contributing 11 (46%) studies of the total, significantly exceeding other regions. Europe followed with 8 (33%) studies, while the Middle East and Oceania contributed 2 (8%) and 3 (13%) studies, respectively. At the country level, the United States emerged as the leading research site, accounting for 10 (42%) studies—the highest among all countries. Spain and Australia each contributed 2 studies. Other countries—namely, Switzerland, Turkey, Germany, Israel, Belgium, New Zealand, Iceland, Canada, the United Kingdom, and Finland—each contributed 1 study. Regional variations were also observed in the selection and application of intervention technologies. North American countries (eg, the United States and Canada) generally favored the combination of wearable devices and mobile apps, providing integrated data tracking and personalized feedback. By contrast, several European countries primarily integrated traditional intervention models with digital platforms, including web-based systems and educational software. Although studies from Middle Eastern and Oceanic countries featured smaller sample sizes, their intervention formats were diverse, incorporating tools such as SMS text messaging and interactive platforms (see Multimedia Appendix 3).

#### Intervention Duration and Frequency

Considerable variation was observed in the duration of interventions. The shortest intervention lasted 1 week (excluding 3 CSSs), while the longest extended to 8 months. The most commonly adopted duration was 12 weeks (3 months), reported in 4 (17%) studies, followed by durations of 6, 8, and 10 weeks, each reported in 2 (8%) studies. Other durations included 1 week, 2 weeks, and 8 months, each reported in a single study (4%). Overall, approximately one-third of the studies fell within the 6- to 12-week range. Based on duration, short-term interventions (≤4 weeks) were reported in 5 (21%) studies; medium-term interventions (5-12 weeks) were the most prevalent, appearing in 11 (46%) studies; and long-term interventions (>12 weeks) were implemented in 5 (21%) studies. Long-term interventions were predominantly associated with high-quality RCTs, whereas short-term interventions were distributed across a variety of study designs. In summary, the majority of studies employed medium-term interventions, typically lasting 2-3 months.

#### Risk of Bias in Studies

Among the 24 included studies, research designs comprised 10 (42%) RCTs, 4 (17%) QES, 3 (13%) QR, 3 (13%) CSS, and 4 (17%) MMS. Quality appraisal results indicated that 7 (29%) studies were rated as high quality, 16 (67%) as moderate quality, and 1 (4%) as low quality. Further analysis revealed that most RCTs achieved high or moderate quality ratings, while QES, CSS, and MMS studies generally met moderate or higher quality standards. Notably, QR studies demonstrated outstanding performance, with 2 projects achieving high quality (see Figure 2).

### Classification and Outcomes of DHIs

The classification and corresponding outcomes of DHIs are presented in Table 2. The types of interventions employed across the included studies exhibited considerable diversity.

These interventions were broadly categorized into 3 types: single-driver interventions, multimodal integrated interventions, and interaction-enhanced interventions. Based on the combined findings from Tables 1 and 2, single-driver interventions were the most commonly reported, appearing in 14 (58%) studies. Interventions in this category typically relied on a single technological medium, most commonly smartphones as the primary device. mHealth interventions were the most prevalent, followed by virtual platform–based interventions and robotic systems delivering personalized SMS text messages. Multimodal integrated interventions were identified in 7 (29%) studies. These interventions combined 2 or more digital components, including mHealth apps, wearable devices, and web-based platforms. Interaction-enhanced interventions were relatively limited, reported in only 3 (13%) studies. Characterized by active adolescent engagement—such as promoting physical activity through video chats—these interventions primarily involved gamified strategies, social media platforms, and SMS text message–based messaging systems.

Overall, all 3 categories of DHIs demonstrated positive outcomes. As shown in Table 2, the majority of studies were classified as “D” (directly effective; 15/24, 63%), while smaller proportions were categorized as “I” (indirectly effective; 8/24, 33%) or “U” (unclear; 1/24, 4%).

Among the “D” interventions, most studies reported statistically significant improvements in physical activity outcomes [19,22-24,26-33,35,37,38], including increased step counts, longer durations of moderate-to-vigorous physical activity, and greater participation in sports. Several studies also reported improvements in physical health indicators related to physical activity [15-18,20,21,25,34], including reductions in BMI, improved body composition and physical fitness, and decreased body fat percentage. Studies classified as “I” did not report direct increases in physical activity levels but demonstrated positive effects on psychological mechanisms, such as enhanced exercise motivation and self-efficacy. These effects included strengthened beliefs in healthy lifestyles, the establishment of exercise goals that promote physical activity, and increased awareness of the benefits of physical activity [36]. Studies categorized as “U” lacked conclusive evidence, making it difficult to determine the effectiveness of the DHIs under investigation.

### Results of Syntheses

A comprehensive analysis of the included studies was conducted based on the form of DHIs and their effectiveness characteristics. The synthesis identified significant variation in research designs (eg, QR, CSS, RCT, QES, MMS), sample sizes (10-9940 participants), outcome measurement methods, and methodological quality. Overall study quality was predominantly moderate to high, with most interventions lasting short to medium durations. As a result of substantial heterogeneity in study design, outcome definitions, and statistical analysis methods, as well as the absence of standardized effect size parameters for pooling across studies, a quantitative meta-analysis was not performed. Instead, results were synthesized using narrative synthesis and qualitative content analysis based on intervention type. The findings are presented in structured tables and categorized descriptions (Tables 1 and 2) to compare the effectiveness of different intervention formats and study designs in promoting physical activity among adolescents.

### Reporting Biases and Certainty of Evidence

As this systematic review did not perform a quantitative meta-analysis and due to considerable variation in study designs and outcome reporting methods, no formal testing for publication bias was conducted using funnel plots or statistical methods. Instead, a qualitative assessment of potential reporting bias was undertaken during evidence synthesis and interpretation. Evidence certainty was not formally assessed using a quantitative tool; rather, the credibility of the evidence was judged by integrating study design type, methodological quality assessment results, and the consistency of study conclusions. Overall, the included studies were of moderate-to-high quality (Figures 1-4). However, owing to study heterogeneity and methodological limitations, the conclusions should be interpreted within the context of specific research settings.

## Discussion

### Principal Findings

This systematic review synthesizes evidence from diverse DHIs and study designs to evaluate their role in promoting physical activity among adolescents and to explore their adaptability across different research designs and settings. Results from the 24 included studies indicate that DHIs can enhance adolescents’ physical activity through direct or indirect mechanisms. Substantial heterogeneity was observed among the studies in terms of research design types, resulting in inconsistent evidence strength and limited generalizability; this heterogeneity represents a key finding of the review. A systematic analysis of intervention formats further revealed that different types of DHIs (single-driver, multimodal integrated, and interaction-enhanced) exhibit distinct effect profiles and are applicable in different contexts. In addition, factors such as target population characteristics and intervention duration significantly influence the interpretation and application of outcomes. Accordingly, this review emphasizes the need for cautious interpretation of research findings, with careful consideration of the scope of inference supported by the study designs and implementation contexts.

This review included 3 QR and 3 CSS. As shown in Tables 1 and 2, the evidence derived from these study types differs. QR evidence reflects the meaning or experience of behaviors or events rather than quantitative measurements [39]. For example, Soltero et al [15] used qualitative methods to assess the feasibility and acceptability of DHIs, identifying potential pathways for promoting physical activity among adolescents with obesity. CSSs measure exposure and outcomes at a single time point, revealing correlations between DHI use, individual characteristics, and physical activity levels [40]. For example, Ng et al [20] examined the association between ownership and use of personal activity trackers and physical activity behaviors. Consequently, this evidence is better suited to suggesting potential mechanisms and real-world usage scenarios rather than to drawing strong causal conclusions.

This review observes the indirect effectiveness of DHIs on adolescent physical activity within QR and CSS evidence. Although such interventions may not immediately lead to significant behavioral changes, these study types more readily capture positive psychological shifts, such as enhanced motivation, increased behavioral intent, strengthened self-efficacy, improved health cognition, or greater interest in participation [41]. Compared with RCTs, QR emphasizes contextual experiences and subjective perceptions, making it more effective in identifying positive psychological shifts, such as heightened motivation. This provides crucial insights into the indirect mechanisms of intervention. CSSs excel at revealing structural relationships between psychological or behavioral characteristics and DHI use at a single time point, offering key clues for understanding these indirect mechanisms.

According to self-determination theory [42], the persistence of adolescent behaviors depends not only on external reminders or incentives but also on the fulfillment of fundamental psychological needs, such as autonomy, competence, and relatedness. Among adolescents in the early stages of behavior change or with unstable motivation, such psychological improvements are particularly important [43] and may gradually translate into behavioral changes through positive experiences, goal identification, and social support [44]. This mechanism of psychological-behavioral conversion helps explain the delayed behavioral improvements observed in some follow-up studies; however, further longitudinal research is needed to validate these findings.

While this evidence provides valuable insights into underlying mechanisms and the overall methodological quality is high, risks of bias remain. Some QR studies did not adequately disclose the researcher’s stance [15-17], and some CSSs did not sufficiently control for confounding factors [18,20]. These issues may compromise the robustness of the findings and therefore warrant cautious interpretation.

QR studies and CSSs have distinct characteristics. The former illuminates indirect effects and underlying mechanisms of DHIs, whereas the latter reveals associations among variables but cannot establish causality. Future research should prioritize high-quality study designs to validate the efficacy and long-term effects of DHIs.

This systematic review included 10 RCTs, which provided evidence with varying focuses. RCTs are considered the gold standard for evaluating intervention effectiveness, as their rigorous design allows for systematic assessment of intervention outcomes over time [45]. The RCTs included in this review involved both general adolescent populations and adolescents with specific health conditions, primarily aiming to validate the feasibility or efficacy of DHIs for these groups [21].

Collectively, these studies indicate that DHIs have potential for promoting physical activity. For populations with specific health conditions, effectiveness often depends more on personalized and sustained support than on technological features [46-48]. Although subgroups share certain commonalities, they also differ in their intervention needs. Commonalities include challenges in behavior change, strong demands for psychological and emotional support, and the need for long-term follow-up and sustained support [10,32,49-53]. Differences are reflected in personalized preferences. Adolescents with attention-deficit/hyperactivity disorder may require more structured and highly interactive tools, whereas cancer survivors may prioritize privacy and self-paced control. Adolescents with obesity or overweight issues may benefit from integrated solutions that combine physical activity, dietary adjustments, and psychological support [54-56]. These differences arise from variations in behavioral mechanisms, cognitive characteristics, and motivational responses across populations. Even with robust technology, failure to align interventions with users’ cognition, motivation, and behavior can hinder effective engagement and behavior change. Future research should prioritize methodologically rigorous RCTs and tailored designs that match population characteristics with intervention protocols to enhance evidence interpretability and real-world applicability.

Of the follow-up studies included in this systematic review, 3 were RCTs [22,23,26]. The findings suggest that the presence or absence of follow-up may introduce bias in assessing DHI outcomes. Follow-up is crucial for evaluating the sustainability and long-term impact of interventions, as extended follow-up periods help identify both enduring effects and potential side effects [57]. For example, Larsen et al [22] reported that a 12-week follow-up showed the intervention promoted diverse physical activities. Similarly, Thompson et al [23] found that personalized SMS text message interventions helped adolescents establish long-term exercise habits, with follow-up feedback further optimizing intervention effectiveness.

In this systematic review, studies with follow-up periods allow assessment of the sustainability of intervention effects, whereas studies without follow-up can evaluate only short-term outcomes. Evidence suggests that a 12-week intervention can yield favorable results for adolescents [23], and such feedback helps refine intervention strategies. RCTs provide relatively strong evidence for this review; however, the limited number of studies primarily supports short-term effects and does not adequately validate intervention sustainability or stability. Consequently, robust evidence for the long-term sustainability of DHIs in real-world settings, such as schools and communities, remains insufficient, which may hinder practical implementation and policy support.

The included RCTs indicate that a 3-month (12-week) intervention duration is the most common. This cycle facilitates gradual behavior establishment and consolidation while minimizing negative effects such as fatigue, a finding widely recognized [23]. Research suggests that shorter intervention durations are insufficient to produce significant changes, whereas excessively long durations may increase complexity and reduce adherence [58]. However, no studies specifically examined the impact of varying intervention durations on adolescent populations.

The impact of sample attrition on outcomes cannot be overlooked. Three of the included studies reporting attrition were RCTs [23,28,30]. Our analysis found that attrition typically occurs in studies requiring sustained participation and long-term engagement, making it a critical factor influencing outcomes. Studies with high attrition rates often face challenges such as insufficient intervention personalization, limited feedback, and inadequate technical support, all of which can weaken participants’ motivation and commitment.

From a behavioral science perspective, based on the COM-B framework, if interventions fail to sustainably enhance individuals’ sense of competence, adolescents are prone to burnout and disengagement. Egilsson et al [28] confirmed this, suggesting that high attrition rates during 6-week follow-ups may be related to insufficient intervention personalization, monotonous formats, or technical barriers. Conversely, other studies found that lower attrition rates are closely associated with adolescent cocreation of intervention content [23]. Thus, enhancing intervention personalization, diversity, and interactivity can reduce attrition, thereby improving research stability and the generalizability of findings.

As shown in Table 2, among the studies demonstrating direct effectiveness, 8 were RCTs. RCTs minimize confounding bias through randomization, which helps clarify intervention effects. Analysis indicates that these interventions promote short-term behavioral changes (eg, increased daily step counts) through goal setting, task execution, and feedback design [59]. This effect depends on clear goals and immediate feedback, enabling adolescents to master behavioral cognition through tracking or outcome comparisons [60]. Such interventions drive strong short-term behavioral change, particularly among participants with initial motivation. The core mechanism enhances individual competence and execution capacity, fosters a sense of accomplishment, and lays the foundation for long-term behavioral strategies.

In summary, RCT designs provided key evidence for this review, although some biases remain. Many studies, due to the nature of DHIs, lacked blinding and faced challenges in follow-up and assessment, which may have affected the reliability of their findings. Future research should employ high-quality RCTs with robust follow-up designs to assess long-term effects and address sample attrition. Given that over half of existing studies used RCTs to evaluate direct effects, future studies should select study designs that align with specific objectives and research contexts.

This systematic review included 4 MMS and 4 QES. MMS combines quantitative and qualitative approaches, enabling simultaneous evaluation of effects and underlying mechanisms, whereas QES assesses impacts but is prone to selection bias due to the absence of randomization or control groups [61]. Given the limited number of MMS and QES, the evidence is scattered. These study types are suitable for identifying feasible strategies and potential mechanisms in real-world contexts, but should not be overgeneralized into universal conclusions. For example, one study from each design type explored the effectiveness and feasibility of DHIs for promoting physical activity in adolescents with specific health conditions. As shown in Table 2, the majority of studies within both research designs demonstrated the direct effectiveness of DHIs, supporting the applicability of these approaches. However, the overall quality was moderate, likely due to small sample sizes and selection bias. Given the limited number of studies, the evidence lacks representativeness and should be interpreted with caution.

Beyond the evidence provided by different study designs, we identified additional relevant findings. Compared with previous systematic reviews [9-11], this review included multiple types of DHIs and categorized them thematically. Results indicate that most studies employed single-driver interventions, while a minority utilized multimodal integrated or interaction-enhanced interventions. Each intervention type exhibits distinct characteristics, tailored to different target populations and intervention phases (see Multimedia Appendix 3 and Table 2).

First, single-driver interventions, such as smartphone apps, promote behavior change through goal setting and reminders and are particularly effective during the initiation phase, leading to significant increases in daily step counts [10,62]. However, their effects are limited by the lack of continuous feedback, restricting their applicability to short-term behavior promotion [63]. Second, multimodal integrated interventions combine wearable devices with mHealth technologies. These interventions are well-suited for the long-term management of high-risk populations, consistently increasing activity frequency and reducing sedentary behavior [64,65]. Nevertheless, their complexity and user burden may hinder acceptance and adherence [66], highlighting the need for simplified workflows to enhance participation.

Third, interaction-enhanced interventions leverage gamification and social interaction to stimulate adolescents’ emotional and social motivation, thereby increasing participation enthusiasm and self-efficacy [67]. Their strength lies in enhancing behavioral appeal and fostering sustained engagement, making them potent drivers of short-term behavioral change. The core mechanism involves strengthening individuals’ sense of competence and execution capacity, enabling them to experience accomplishment and receive positive feedback upon goal attainment, thereby laying the foundation for long-term behavioral strategies. However, effectiveness may vary among individuals with weaker social motivation or limited digital access [32,49]. Current research predominantly focuses on single-driver interventions due to their operational simplicity. Nonetheless, distinct characteristics and limitations emerge across intervention types when applied to diverse adolescent groups and developmental stages. Consequently, the selection of intervention type can significantly influence outcomes, particularly for specific populations and developmental phases.

On a broader scale, this systematic review highlights 2 critical real-world challenges in DHIs. First, these interventions reflect regional disparities and health inequalities [68]. North America, particularly the United States, leads in research and technological applications, emphasizing personalized feedback and data tracking [69,70]. In Europe, although fewer studies are conducted, there is a focus on educational and universal approaches tailored to diverse sociocultural contexts [71]. Meanwhile, the Middle East and Oceania explore a variety of technological pathways within the constraints of limited resources [72].

However, these regional disparities not only reflect differences in technological adoption but also highlight digital health inequalities across domains. Digital health disparities are becoming increasingly prominent worldwide, particularly in low- and middle-income countries [73], where significant gaps persist in the accessibility and effectiveness of DHIs. These disparities are largely influenced by factors such as infrastructure development and digital literacy [74]. Therefore, in promoting global DHIs, greater focus must be placed on addressing these regional inequalities. Measures should be taken to allocate resources appropriately and enhance technical support to ensure the inclusivity and equity of technology.

Second, the technological dependency of DHIs is both a crucial factor influencing outcomes and a determinant of long-term adherence. When interventions rely on digital platforms, apps, or wearable devices, participants’ sustained engagement may be disrupted by technical issues, thereby affecting intervention stability and the assessment of outcomes [75-77].

Mechanistically, the effectiveness of system design in technological interventions largely determines participants’ long-term adherence, as supported by previous studies. For example, Jakob et al [78] found that sustained use of mHealth apps is influenced by design factors such as technical stability and personalized push notifications. Similarly, Kelders et al [79] demonstrated that digital interventions incorporating persuasive design significantly enhance user adherence. Therefore, addressing the negative impacts of technological dependency represents a critical future development pathway for DHIs.

### Limitations

This systematic review has several key limitations. First, the included studies exhibited considerable heterogeneity in design type, intervention format, feedback mechanisms, duration, and behavioral assessment methods. This heterogeneity limited evidence consistency and hindered the integration and generalizability of the findings. Second, many original studies had brief evaluation periods and lacked medium- to long-term follow-up data, complicating the assessment of intervention sustainability and long-term impact. Additionally, some studies had methodological limitations and insufficient reporting transparency. Although most studies were of moderate to high quality, these issues compromised the reliability and generalizability of the evidence. Finally, despite conducting multidatabase systematic searches, relevant literature may have been omitted due to limitations in search coverage and potential publication bias.

Therefore, future research can be enhanced in several areas. First, the study design should be strengthened to elevate overall evidence quality. Conducting more high-quality RCTs would improve the scientific rigor and feasibility of intervention studies. Standardization of intervention content and behavioral indicators should be promoted, establishing a unified assessment system to enhance comparability and integration of results across studies. Simultaneously, methodological design and reporting transparency should be improved to provide a more reliable evidence base for systematic reviews.

Second, future research should integrate discussions of intervention mechanisms and effects into study methodologies. The subjective experiences and underlying mechanisms of DHIs hold significant research value. Combining subjective experience data with objective behavioral data can generate more comprehensive and representative evidence. A deeper understanding of the transformation processes underlying these mechanisms will provide a foundation for developing personalized intervention models. Finally, continuous research should address practical issues, such as strengthening follow-up. Study designs should incorporate more follow-up components to systematically document postintervention behavioral maintenance and motivational changes. Concurrently, attention should be given to populations with specific health conditions, digital health inequalities, and technology dependency challenges, to enhance the interpretive power and practical applicability of research in real-world contexts.

### Conclusions

Against the backdrop of increasing physical inactivity and sedentary behavior among adolescents globally, this systematic review synthesizes evidence from 24 studies encompassing diverse research designs and multiple types of DHIs. Compared with previous systematic reviews that focused exclusively on specific study designs or single technological formats, this review provides a more holistic and comparative perspective on emerging evidence. The integrated findings indicate that DHIs generally contribute to enhancing adolescents’ physical activity levels. However, their effectiveness varies considerably across intervention types, research designs, and application contexts. This finding addresses gaps in existing research, suggesting that both the adaptability of interventions and the implementation setting play crucial roles in shaping outcomes. Distinct strengths were observed across different research designs: RCTs provided relatively reliable evidence for direct and long-term intervention effects, while QR and CSS contributed to understanding underlying mechanisms and associations. QES and some MMS offered relatively limited representativeness due to scattered evidence. This finding supplements and refines the existing understanding of the evidence structure for DHIs. Further analysis indicates that the suitability of different DHIs varies across adolescent populations and practice settings, with the choice of intervention type significantly influencing outcomes. Concurrent attention to adolescents with specific health conditions, digital health inequities, and technology dependency challenges provides new perspectives for equitable implementation and sustainable scaling of DHIs in real-world settings. Despite limitations in the existing evidence—including methodological bias, inconsistent study quality, and insufficient follow-up data—the findings of this review remain valuable for understanding the role of DHIs in promoting adolescent physical activity. They provide useful insights for practical application, global health promotion, health equity, and innovation in cross-cultural intervention strategies. Future research should integrate the strengths of diverse study designs to conduct high-quality, long-term follow-up studies. Such studies should comprehensively consider target population characteristics, intervention duration, and regional variations to enhance the effectiveness and scalability of DHIs in real-world settings.

## Appendix

Multimedia Appendix 1 Search strategy.

Multimedia Appendix 2 PRISMA (Preferred Reporting Items for Systematic Reviews and Meta-Analyses) checklist.

Multimedia Appendix 3 Additional analysis.

## Abbreviations

BCW: Behavior Change Wheel
COM-B: capability, opportunity, motivation—behavior
CSS: cross-sectional study
DHI: digital health intervention
GRADE: Grading of Recommendations, Assessment, Development and Evaluation
JBI: Joanna Briggs Institute
MeSH: Medical Subject Headings
mHealth: mobile health
MMS: mixed methods study
PICOS: population, intervention, comparator, outcomes, and study design
PRISMA: Preferred Reporting Items for Systematic Reviews and Meta-Analyses
PRISMA-S: Preferred Reporting Items for Systematic Reviews and Meta-Analyses—Search Extension
PROSPERO: International Prospective Register of Systematic Reviews
QES: quasi-experimental study
QR: quantitative research
RCT: randomized controlled trial

## Data Availability

All data generated or analyzed during this systematic review are included in this published article and its multimedia appendices. Additional information is available from the corresponding author (LJ) on reasonable request.

## References

[1] Chaput J, Willumsen J, Bull F, Chou R, Ekelund U, Firth J, Jago R, Ortega FB, Katzmarzyk PT (2020). 2020 WHO guidelines on physical activity and sedentary behaviour for children and adolescents aged 5-17 years: summary of the evidence. Int J Behav Nutr Phys Act.

[2] Rose T, Barker M, Maria Jacob C, Morrison L, Lawrence W, Strömmer Sofia, Vogel C, Woods-Townsend K, Farrell D, Inskip H, Baird J (2017). A systematic review of digital interventions for improving the diet and physical activity behaviors of adolescents. J Adolesc Health.

[3] Sampasa-Kanyinga H, Colman I, Goldfield GS, Janssen I, Wang J, Podinic I, Tremblay MS, Saunders TJ, Sampson M, Chaput J (2020). Combinations of physical activity, sedentary time, and sleep duration and their associations with depressive symptoms and other mental health problems in children and adolescents: a systematic review. Int J Behav Nutr Phys Act.

[4] Düking Peter, Tafler M, Wallmann-Sperlich B, Sperlich B, Kleih S (2020). Behavior change techniques in wrist-worn wearables to promote physical activity: content analysis. JMIR Mhealth Uhealth.

[5] Au WW, Recchia F, Fong DY, Wong SHS, Chan DKC, Capio CM, Yu CCW, Wong SWS, Sit CHP, Ip P, Chen Y, Thompson WR, Siu PM (2024). Effect of wearable activity trackers on physical activity in children and adolescents: a systematic review and meta-analysis. Lancet Digit Health.

[6] Michie S, van Stralen MM, West R (2011). The behaviour change wheel: a new method for characterising and designing behaviour change interventions. Implement Sci.

[7] Collin C, Eyraud C, Martin P, Michel M, Le Roux E, Alberti C (2025). A scoping review of outcome selection and accuracy of conclusions in complex digital health interventions for young people (2017-2023): methodological proposals for population health intervention research. BMC Med.

[8] Partridge SR, Knight A, Todd A, McGill B, Wardak S, Alston L, Livingstone KM, Singleton A, Thornton L, Jia S, Redfern J, Raeside R (2024). Addressing disparities: a systematic review of digital health equity for adolescent obesity prevention and management interventions. Obes Rev.

[9] Wang J, Zhu Z, Shuling Z, Fan J, Jin Y, Gao Z, Chen W, Li X (2024). Effectiveness of mHealth app-based interventions for increasing physical activity and improving physical fitness in children and adolescents: systematic review and meta-analysis. JMIR Mhealth Uhealth.

[10] He Z, Wu H, Yu F, Fu J, Sun S, Huang T, Wang R, Chen D, Zhao G, Quan M (2021). Effects of smartphone-based interventions on physical activity in children and adolescents: systematic review and meta-analysis. JMIR Mhealth Uhealth.

[11] Sequí-Domínguez Irene, Cavero-Redondo I, Álvarez-Bueno Celia, López-Gil Jose Francisco, Martínez-Vizcaíno Vicente, Pascual-Morena C (2024). Effectiveness of eHealth interventions promoting physical activity in children and adolescents: systematic review and meta-analysis. J Med Internet Res.

[12] Page MJ, McKenzie JE, Bossuyt PM, Boutron I, Hoffmann TC, Mulrow CD, Shamseer L, Tetzlaff JM, Akl EA, Brennan SE, Chou R, Glanville J, Grimshaw JM, Hróbjartsson Asbjørn, Lalu MM, Li T, Loder EW, Mayo-Wilson E, McDonald S, McGuinness LA, Stewart LA, Thomas J, Tricco AC, Welch VA, Whiting P, Moher D (2021). The PRISMA 2020 statement: an updated guideline for reporting systematic reviews. Rev Esp Cardiol (Engl Ed).

[13] Barker T, Stone J, Sears K, Klugar M, Tufanaru C, Leonardi-Bee J, Aromataris E, Munn Z (2023). The revised JBI critical appraisal tool for the assessment of risk of bias for randomized controlled trials. JBI Evid Synth.

[14] Rethlefsen ML, Kirtley S, Waffenschmidt S, Ayala AP, Moher D, Page MJ, Koffel JB, PRISMA-S Group (2021). PRISMA-S: an extension to the PRISMA statement for reporting literature searches in systematic reviews. Syst Rev.

[15] Soltero EG, Musaad SM, O'Connor Teresia M, Thompson D, Norris K, Beech BM (2024). Feasibility of Fit24, a digital diabetes prevention program for Hispanic adolescents: qualitative evaluation study. JMIR Form Res.

[16] Goodyear VA, Kerner C, Quennerstedt M (2017). Young people’s uses of wearable healthy lifestyle technologies; surveillance, self-surveillance and resistance. Sport, Education and Society.

[17] Van Dyck D, D'Haese Sara, Plaete J, De Bourdeaudhuij I, Deforche B, Cardon G (2019). Opinions towards physical activity interventions using Facebook or text messaging: focus group interviews with vocational school-aged adolescents. Health Soc Care Community.

[18] Dinç Fatma, Kurt A, Yıldız D (2025). The use of mobile health applications in the development of a healthy lifestyle of adolescents: a cross-sectional study. J Pediatr Nurs.

[19] Mojica CM, Parra-Medina D, Yin Z, Akopian D, Esparza LA (2014). Assessing media access and use among Latina adolescents to inform development of a physical activity promotion intervention incorporating text messaging. Health Promot Pract.

[20] Ng Kwok, Kokko Sami, Tammelin Tuija, Kallio Jouni, Belton Sarahjane, O'Brien Wesley, Murphy Marie, Powell Cormac, Woods Catherine (2020). Clusters of Adolescent Physical Activity Tracker Patterns and Their Associations With Physical Activity Behaviors in Finland and Ireland: Cross-Sectional Study. J Med Internet Res.

[21] Mendoza JA, Baker KS, Moreno MA, Whitlock K, Abbey-Lambertz Mark, Waite A, Colburn T, Chow EJ (2017). A Fitbit and Facebook mHealth intervention for promoting physical activity among adolescent and young adult childhood cancer survivors: a pilot study. Pediatr Blood Cancer.

[22] Larsen B, Benitez T, Cano M, Dunsiger SS, Marcus BH, Mendoza-Vasconez A, Sallis JF, Zive M (2018). Web-based physical activity intervention for Latina adolescents: feasibility, acceptability, and potential efficacy of the Niñas Saludables study. J Med Internet Res.

[23] Thompson D, Cantu D, Ramirez B, Cullen KW, Baranowski T, Mendoza J, Anderson B, Jago R, Rodgers W, Liu Y (2016). Texting to increase adolescent physical activity: feasibility assessment. Am J Health Behav.

[24] Guthrie N, Bradlyn A, Thompson SK, Yen S, Haritatos J, Dillon F, Cole SW (2015). Development of an accelerometer-linked online intervention system to promote physical activity in adolescents. PLoS One.

[25] Direito A, Jiang Y, Whittaker R, Maddison R (2015). Smartphone apps to improve fitness and increase physical activity among young people: protocol of the Apps for IMproving FITness (AIMFIT) randomized controlled trial. BMC Public Health.

[26] Chen J, Guedes CM, Cooper BA, Lung AE (2017). Short-term efficacy of an innovative mobile phone technology-based intervention for weight management for overweight and obese adolescents: pilot study. Interact J Med Res.

[27] Caillaud C, Ledger S, Diaz C, Clerc G, Galy O, Yacef K (2022). iEngage: a digital health education program designed to enhance physical activity in young adolescents. PLoS One.

[28] Egilsson E, Bjarnason R, Njardvik U (2021). Usage and weekly attrition in a smartphone-based health behavior intervention for adolescents: pilot randomized controlled trial. JMIR Form Res.

[29] Staiano AE, Beyl RA, Guan W, Hendrick CA, Hsia DS, Newton RL (2018). Home-based exergaming among children with overweight and obesity: a randomized clinical trial. Pediatr Obes.

[30] Stasinaki A, Büchter D, Shih CI, Heldt K, Güsewell S, Brogle B, Farpour-Lambert N, Kowatsch T, l'Allemand D (2021). Effects of a novel mobile health intervention compared to a multi-component behaviour changing program on body mass index, physical capacities and stress parameters in adolescents with obesity: a randomized controlled trial. BMC Pediatr.

[31] Ortega A, Cushing CC (2024). Design of a temporally augmented text messaging bot to improve adolescents' physical activity and engagement: proof-of-concept study. JMIR Form Res.

[32] Willinger L, Schweizer F, Böhm Birgit, Scheller DA, Jonas S, Oberhoffer-Fritz R, Müller Jan, Reimer LM (2024). Evaluation of the gamified application KIJANI to promote physical activity in children and adolescents: a multimethod study. Digit Health.

[33] Schoenfelder E, Moreno M, Wilner M, Whitlock KB, Mendoza JA (2017). Piloting a mobile health intervention to increase physical activity for adolescents with ADHD. Prev Med Rep.

[34] Koorts H, Salmon J, Timperio A, Ball K, Macfarlane S, Lai SK, Brown H, Chappel SE, Lewis M, Ridgers ND (2020). Translatability of a wearable technology intervention to increase adolescent physical activity: mixed methods implementation evaluation. J Med Internet Res.

[35] Glaser M, Green G, Barak S, Bord S, Levi S, Jakobovich R, Dunsky A, Zigdon A, Zwilling M, Tesler R (2024). The effects of the Friendship Online Intervention Program on physical activity, substance abuse, psychosomatic symptoms, and well-being among at-risk youth. J Adolesc.

[36] Garde A, Umedaly A, Abulnaga SM, Robertson L, Junker A, Chanoine JP, Ansermino JM, Dumont GA (2015). Assessment of a mobile game ("MobileKids Monster Manor") to promote physical activity among children. Games Health J.

[37] Mateo-Orcajada A, Abenza-Cano L, Albaladejo-Saura MD, Vaquero-Cristóbal Raquel (2023). Mandatory after-school use of step tracker apps improves physical activity, body composition and fitness of adolescents. Educ Inf Technol (Dordr).

[38] Cushing C, Bejarano C, Ortega A, Sayre N, Fedele D, Smyth J (2021). Adaptive mHealth intervention for adolescent physical activity promotion. J Pediatr Psychol.

[39] Moffatt S, White M, Mackintosh J, Howel D (2006). Using quantitative and qualitative data in health services research - what happens when mixed method findings conflict? [ISRCTN61522618]. BMC Health Serv Res.

[40] Wang X, Cheng Z (2020). Cross-sectional studies: strengths, weaknesses, and recommendations. Chest.

[41] Domhardt M, Schröder Annalena, Geirhos A, Steubl L, Baumeister H (2021). Efficacy of digital health interventions in youth with chronic medical conditions: a meta-analysis. Internet Interv.

[42] Ryan RM, Deci EL (2000). Self-determination theory and the facilitation of intrinsic motivation, social development, and well-being. Am Psychol.

[43] Zhang G, Feng W, Zhao L, Zhao X, Li T (2024). The association between physical activity, self-efficacy, stress self-management and mental health among adolescents. Sci Rep.

[44] Teixeira PJ, Silva MN, Mata J, Palmeira AL, Markland D (2012). Motivation, self-determination, and long-term weight control. Int J Behav Nutr Phys Act.

[45] Collin Claire, Eyraud Clara, Martin Philippe, Michel Morgane, Le Roux Enora, Alberti Corinne (2025). A scoping review of outcome selection and accuracy of conclusions in complex digital health interventions for young people (2017-2023): methodological proposals for population health intervention research. BMC Med.

[46] Stavric V, Kayes NM, Rashid U, Saywell NL (2022). The effectiveness of self-guided digital interventions to improve physical activity and exercise outcomes for people with chronic conditions: a systematic review and meta-analysis. Front Rehabil Sci.

[47] Michalsen H, Wangberg SC, Hartvigsen G, Jaccheri L, Muzny M, Henriksen A, Olsen MI, Thrane G, Jahnsen RB, Pettersen G, Arntzen C, Anke A (2020). Physical activity with tailored mHealth support for individuals with intellectual disabilities: protocol for a randomized controlled trial. JMIR Res Protoc.

[48] Ma J, Floegel T, Li L, Leese J, De Vera Mary A, Beauchamp M, Taunton J, Liu-Ambrose T, Allen K (2021). Tailored physical activity behavior change interventions: challenges and opportunities. Transl Behav Med.

[49] Shameli A, Althoff T, Saberi A, Leskovec J (2017). How gamification affects physical activity: large-scale analysis of walking challenges in a mobile application. Proc Int World Wide Web Conf.

[50] Bandura A (1997). Self-Efficacy: The Exercise of Control.

[51] Flølo Tone Nygaard, Tørris Christine, Riiser K, Almendingen K, Chew HSJ, Fosså Alexander, Albertini Früh Elena, Hennessy E, Leung MM, Misvær Nina, Pavel N, Sundar TKB, Sæterstrand Toril Margaret, Torbjørnsen Astrid, Løyland Borghild, Holmen H (2025). Digital health interventions to treat overweight and obesity in children and adolescents: an umbrella review. Obes Rev.

[52] van Baak MA, Pramono A, Battista F, Beaulieu K, Blundell JE, Busetto L, Carraça Eliana V, Dicker D, Encantado J, Ermolao A, Farpour-Lambert Nathalie, Woodward E, Bellicha A, Oppert J (2021). Effect of different types of regular exercise on physical fitness in adults with overweight or obesity: systematic review and meta-analyses. Obes Rev.

[53] Fismen A, Galler M, Klepp K, Chatelan A, Residori C, Ojala K, Dzielska A, Kelly C, Melkumova M, Musić Milanović S, Nardone P, Štefanová Eliška, Flodgren G, Bakke T, Ercan O, Samdal O, Helleve A (2022). Weight status and mental well-being among adolescents: the mediating role of self-perceived body weight. a cross-national survey. J Adolesc Health.

[54] Kollins SH, DeLoss DJ, Cañadas Elena, Lutz J, Findling RL, Keefe RSE, Epstein JN, Cutler AJ, Faraone SV (2020). A novel digital intervention for actively reducing severity of paediatric ADHD (STARS-ADHD): a randomised controlled trial. Lancet Digit Health.

[55] Wang W, Cheng J, Song W, Shen Y (2022). The effectiveness of wearable devices as physical activity interventions for preventing and treating obesity in children and adolescents: systematic review and meta-analysis. JMIR Mhealth Uhealth.

[56] Stamatis CA, Farlow DN, Mercaldi C, Suh M, Maple A, Savarese A, Childress A, Melmed RD, Kollins SH (2024). Two single arm trials of AKL-T01, a digital therapeutic for adolescents and adults with ADHD. Npj Ment Health Res.

[57] Cuzick J (2023). The importance of long-term follow up of participants in clinical trials. Br J Cancer.

[58] Kahwati L, Viswanathan M, Golin CE, Kane H, Lewis M, Jacobs S (2016). Identifying configurations of behavior change techniques in effective medication adherence interventions: a qualitative comparative analysis. Syst Rev.

[59] Bi S, Yuan J, Wang Y, Zhang W, Zhang L, Zhang Y, Zhu R, Luo L (2024). Effectiveness of digital health interventions in promoting physical activity among college students: systematic review and meta-analysis. J Med Internet Res.

[60] Kassim PSJ, Muhammad NA, Rahman NFA, Sidik SM, Essau CA, Shah SA (2022). Digital behaviour change interventions to promote physical activity in overweight and obese adolescents: a systematic review protocol. Syst Rev.

[61] Barker T, Habibi N, Aromataris E, Stone J, Leonardi-Bee J, Sears K, Hasanoff S, Klugar M, Tufanaru C, Moola S, Munn Z (2024). The revised JBI critical appraisal tool for the assessment of risk of bias for quasi-experimental studies. JBI Evid Synth.

[62] Gómez-Cuesta Nerea, Mateo-Orcajada A, Meroño Lourdes, Abenza-Cano L, Vaquero-Cristóbal Raquel (2024). A mobile app-based intervention improves anthropometry, body composition and fitness, regardless of previous active-inactive status: a randomized controlled trial. Front Public Health.

[63] Silva AG, Simões Patrícia, Queirós Alexandra, P Rocha N, Rodrigues M (2020). Effectiveness of mobile applications running on smartphones to promote physical activity: a systematic review with meta-analysis. Int J Environ Res Public Health.

[64] Howard-Wilson S, Ching J, Gentile S, Ho M, Garcia A, Ayturk D, Lazar P, Hammerquist N, McManus D, Barton B, Bird S, Moore J, Soni A (2024). Efficacy of a multimodal digital behavior change intervention on lifestyle behavior, cardiometabolic biomarkers, and medical expenditure: protocol for a randomized controlled trial. JMIR Res Protoc.

[65] Böhm Birgit, Karwiese SD, Böhm Harald, Oberhoffer R (2019). Effects of mobile health including wearable activity trackers to increase physical activity outcomes among healthy children and adolescents: systematic review. JMIR Mhealth Uhealth.

[66] Singh B, Ahmed M, Staiano AE, Gough C, Petersen J, Vandelanotte C, Kracht C, Huong C, Yin Z, Vasiloglou MF, Pan C, Short CE, Mclaughlin M, von Klinggraeff L, Pfledderer CD, Moran LJ, Button AM, Maher CA (2024). A systematic umbrella review and meta-meta-analysis of eHealth and mHealth interventions for improving lifestyle behaviours. NPJ Digit Med.

[67] Bas-Sarmiento P, Julián-López C, Fernández-Gutiérrez M, Poza-Méndez M, Marín-Paz A (2025). Gamified eHealth interventions for health promotion and disease prevention in children and adolescents: a scoping review. Humanit Soc Sci Commun.

[68] Badr J, Motulsky A, Denis J (2024). Digital health technologies and inequalities: a scoping review of potential impacts and policy recommendations. Health Policy.

[69] Ghadi YY, Shah SFA, Waheed W, Mazhar T, Ahmad W, Saeed MM, Hamam H (2025). Integration of wearable technology and artificial intelligence in digital health for remote patient care. J Cloud Comp.

[70] Grand VR, Inc (2024). U.S. Digital Health Market Size, Share. Grand View Research.

[71] Majcherek D, Hegerty SW, Kowalski AM, Lewandowska MS, Dikova D (2024). Opportunities for healthcare digitalization in Europe: comparative analysis of inequalities in access to medical services. Health Policy.

[72] El-Jardali F, Bou-Karroum L, Jabbour M, Bou-Karroum K, Aoun A, Salameh S, Mecheal P, Sinha C (2023). Digital health in fragile states in the Middle East and North Africa (MENA) region: a scoping review of the literature. PLoS One.

[73] Sylla B, Ismaila O, Diallo G (2025). 25 years of digital health toward universal health coverage in low- and middle-income countries: rapid systematic review. J Med Internet Res.

[74] (2024). ew GSMA report shows mobile internet connectivity continues to grow globally but barriers for 3.45 billion unconnected people remain. GSMA.

[75] Linardon J, Fuller-Tyszkiewicz M (2020). Attrition and adherence in smartphone-delivered interventions for mental health problems: a systematic and meta-analytic review. J Consult Clin Psychol.

[76] Pratap A, Neto EC, Snyder P, Stepnowsky C, Elhadad N, Grant D, Mohebbi MH, Mooney S, Suver C, Wilbanks J, Mangravite L, Heagerty PJ, Areán Pat, Omberg L (2020). Indicators of retention in remote digital health studies: a cross-study evaluation of 100,000 participants. NPJ Digit Med.

[77] Pathiravasan CH, Zhang Y, Wang X, Trinquart L, Benjamin EJ, Borrelli B, McManus DD, Kheterpal V, Lin H, Spartano NL, Schramm E, Liu C, Murabito JM (2022). Factors associated with long-term use of digital devices in the electronic Framingham Heart Study. NPJ Digit Med.

[78] Jakob R, Harperink S, Rudolf AM, Fleisch E, Haug S, Mair JL, Salamanca-Sanabria A, Kowatsch T (2022). Factors influencing adherence to mHealth apps for prevention or management of noncommunicable diseases: systematic review. J Med Internet Res.

[79] Kelders SM, Kok RN, Ossebaard HC, Van Gemert-Pijnen JE (2012). Persuasive system design does matter: a systematic review of adherence to web-based interventions. J Med Internet Res.

