# Supplementary material for: Digital Health Interventions to Promote Physical Activity Among Adolescents: Systematic Review
Source: J Med Internet Res. 2026 Feb 27;28:e82395. doi: 10.2196/82395 (PMC13148130; doi:10.2196/82395)
Supplement: Multimedia Appendix 1 [file jmir_v28i1e82395_app1.docx]

***Multimedia Appendix 1 - Search Strategy***

**1** The final keywords chain used in the systematic literature search in **PubMed** were as follows(Used the following Filters: English, all open to access,article published from January 1, 2014, to June 30, 2025.):

**Initial Search Date:**2025.6.10

**Last Search Date:**2025.8.3

**Pub Med** **(via National Library of Medicine platform) Number of records retrieved**:**518**

**#1** "Exercise"[Mesh]

**#2** Physical Activity [tw] OR Exercise [tw] OR Physical fit*[tw] OR Sport* participation[tw] OR Sport* activit* [tw] OR Active lifestyle[tw] OR Movement behavio*[tw] OR MVPA[tw] OR Moderate to vigorous physical activity [tw] OR Step count* [tw] OR Energy expenditur*[tw] OR Daily activit* [tw] OR Walk* [tw] OR Jog[tw] OR Run

**#3** #1 OR #2

**#4** "Digital Health"[Mesh]

**#5** "wearable electronic device"[Mesh]

**#6** "Mobile Applicat"[Mesh]

**#7** "Telemedicine"[Mesh]

**#8** Digital health[tw] OR Mobile health[tw]OR Electronic health[tw] OR eHealth[tw]OR mHealth [tw]OR Wearable device*[tw] OR Fitness track*[tw] OR Fitness tracking device [tw]OR Activity monitor* [tw]OR Smartphone app* [tw]OR Mobile applicat*[tw] OR Mobile phone app[tw] OR Technology-based intervention [tw]OR Virtual fitness platform [tw]OR Online health intervention [tw]OR Digital intervention [tw]OR Gamified health[tw] OR Health gamification [tw]OR Exergame [tw]OR Active video game [tw]OR Internet-based intervention[tw] OR Text message intervention[tw] OR SMS-based intervention [tw]OR Social media intervention*[tw] OR Digital behavior change intervention[tw]

**#9** #4 OR #5 OR #6 OR #7 OR #8

**#10** "Adolescent"[Mesh]

**#11** Adolescen* [tw] OR Teenag* [tw] OR Secondary school student [tw] OR High school student[tw] OR Middle school student [tw] OR School-age* child*[tw]

**#12** #10 OR #11

**#13** #3 AND #9 AND #12

**2** The final keywords chain used in the systematic literature search in **Web of Science**

were as follows(Used the following Filters: English, all open to access,article published from January 1, 2014, to June 30, 2025.):

**Initial Search Date:**2025.6.10

**Last Search Date:**2025.8.3

**Web of Science** **Core Collection (via Clarivate Analytics) Number of records retrieved**:**601**

**#1** TS=(Exercise OR Physical Activity OR Physical fit* OR Sport* participation OR Sport* activit* OR Active lifestyle OR Movement behavio* OR MVPA OR Moderate to vigorous physical activity OR Step count* OR Energy expenditur* OR Daily activit* OR Walk* OR Jog* OR Run*)

**#2** TS=(Digital Health OR wearable electronic device* OR Mobile Applicat* OR Telemedicine OR Mobile health OR Electronic health OR eHealth OR mHealth OR Wearable device* OR Fitness track* OR Fitness tracking device OR Activity monitor* OR Smartphone app* OR Mobile phone app OR Technology-based intervention OR Virtual fitness platform OR Online health intervention OR Digital intervention OR Gamified health OR Health gamification OR Exergame OR Active video game OR Internet-based intervention OR Text message intervention OR SMS-based intervention OR Social media intervention* OR Digital behavior change intervention)

**#3** TS=(Adolescen* OR Teenag* OR Secondary school student OR High school student OR Middle school student OR School-age* child*)

**#4** #1 AND #2 AND #3 AND

**3** The final keywords chain used in the systematic literature search in **EBSCO** were as follows(Used the following Filters: English, all open to access,article published from January 1, 2014, to June 30, 2025.):

**Initial Search Date:**2025.6.10

**Last Search Date:**2025.8.3

**EBSCO** **(via EBSCOhost platform)** **Number of records retrieved**:**50**

**#3** Query (expanded/display term): (SU (Adolescent) OR (Adolescent* OR Teenag* OR "Secondary school student" OR "High school student" OR "Middle school student" OR "School-age* children))

AND

**#2** (SU (Digital Health) OR SU (wearable electronic device*) OR SU (Mobile Applicat*) OR SU (Telemedicine) OR ("Digital health" OR "Mobile health" OR "Electronic health" OR eHealth OR mHealth OR "Wearable device*" OR "Fitness track*" OR "Fitness tracking device" OR "Activity monitor*" OR "Smartphone app*" OR " Mobile applicat*" OR "Mobile phone app" OR "Technology-based intervention" OR "Virtual fitness platform" OR "Online health intervention" OR "Digital intervention" OR "Gamified health" OR "Health gamification" OR Exergame OR "Active video game" OR "Internet-based intervention" OR "Text message intervention" OR "SMS-based intervention" OR "Social media intervention*" OR "Digital behavior change intervention"))

AND

**#1** (SU (Exercise) OR ("Physical Activity" or Exercise OR "Physical fit" OR "Sport* participation" OR "Sport* activit*" OR "Active lifestyle" OR "Movement behavio*" OR MVPA OR "Moderate to vigorous physical activity" OR "Step count*" OR "Energy expenditur*" OR "Daily activit*" OR Walk* OR Jog OR Run))

**Query:** #3 AND #2 AND #1

**4** The final keywords chain used in the systematic literature search in **Scopus** were as follows(Used the following Filters: English, all open to access,article published from January 1, 2014, to June 30, 2025.):

**Initial Search Date:**2025.6.10

**Last Search Date:**2025.8.3

**Scopus** **(via Elsevier platform) Number of records retrieved**:**305**

**#1**TITLE-ABS-KEY ( Exercise OR "Physical Activity" OR "Physical fit*" OR "Sport* participation" OR "Sport* activit*" OR "Active lifestyle" OR "Movement behavio*" OR MVPA OR "Moderate to vigorous physical activity" OR "Step count*" OR "Energy expenditur*" OR "Daily activit*" OR Walk* OR Jog OR Run )

**#2** TITLE-ABS-KEY ( "Digital Health" OR "wearable electronic device*" OR "Mobile Applicat*" OR Telemedicine OR "Mobile health" OR "Electronic health" OR eHealth OR mHealth OR "Wearable device*" OR "Fitness track*" OR "Fitness tracking device" OR "Activity monitor*" OR "Smartphone app*" OR "Mobile phone app" OR "Technology-based intervention" OR "Virtual fitness platform" OR "Online health intervention" OR "Digital intervention" OR "Gamified health" OR "Health gamification" OR Exergame OR "Active video game" OR "Internet-based intervention" OR "Text message intervention" OR "SMS-based intervention" OR "Social media intervention*" OR "Digital behavior change intervention" )

**#3** TITLE-ABS-KEY ( Adolescent* OR Teenag* OR "Secondary school student" OR "High school student" OR "Middle school student" OR "School-age* child*" )

**#4** #1 AND #2 AND #3

**5** The final keywords chain used in the systematic literature search in **Embase** were as follows(Used the following Filters: English, all open to access,,article published from January 1, 2014, to June 30, 2025.):

**Initial Search Date:**2025.6.10

**Last Search Date:**2025.8.3

**Embase (via Elsevier platform)** **Number of records retrieved**:**164**

**#1** 'Exercise'/exp

**#2** 'Physical Activity':ab,ti OR 'Exercise':ab,ti OR 'Physical fit*':ab,ti OR 'Sport* participation':ab,ti OR 'Sport* activit*' :ab,ti OR 'Active lifestyle':ab,ti OR 'Movement behavio*':ab,ti OR 'MVPA':ab,ti OR 'Moderate to vigorous physical activity':ab,ti OR 'Step count*':ab,ti OR 'Energy expenditur*':ab,ti OR 'Daily activit*':ab,ti OR 'Walk*':ab,ti OR 'Jog*':ab,ti OR 'Run*':ab,ti

**#3** #1 OR #2

**#4** 'Digital Health'/exp

**#5** 'Wearable Electronic Devices'/exp

**#6** 'Mobile Applications'/exp

**#7** 'Telemedicine'/exp

**#8** 'Digital health':ab,ti OR 'Mobile health':ab,ti OR 'Electronic health':ab,ti OR 'eHealth':ab,ti OR 'mHealth' :ab,ti OR 'Wearable device*':ab,ti OR 'Fitness track*':ab,ti OR 'Fitness tracking device':ab,ti OR 'Activity monitor*':ab,ti OR 'Smartphone app*':ab,ti OR 'Mobile applicat*':ab,ti OR 'Mobile phone app':ab,ti OR 'Technology-based intervention':ab,ti OR 'Virtual fitness platform':ab,ti OR 'Online health intervention':ab,ti OR 'Digital intervention':ab,ti OR 'Gamified health':ab,ti OR 'Health gamification':ab,ti OR 'Exergame':ab,ti OR 'Active video game':ab,ti OR 'Internet-based intervention':ab,ti OR 'Text message intervention':ab,ti OR 'SMS-based intervention':ab,ti OR 'Social media intervention*':ab,ti OR 'Digital behavior change intervention':ab,ti

**#9** #3 OR #4 OR#5 OR#6 OR#7 OR#8

**#10** 'Adolescent'/exp

**#11** 'Adolescent*':ab,ti OR 'Teenag*':ab,ti OR 'Secondary school student':ab,ti OR 'High school student':ab,ti OR 'Middle school student':ab,ti

**#12** #10 OR #11

**#13** #3 AND #9 AND #12

**6** The final keywords chain used in the systematic literature search in **Cochrane Library** were as follows(Used the following Filters: English, all open to access,,article published from January 1, 2014, to June 30, 2025.):

**Initial Search Date:**2025.6.22

**Last Search Date:**2025.8.3

**Cochrane Library** **(via Wiley Online Library ) Number of records retrieved**:**128**

**#1** MeSH descriptor: [Exercise] explode all trees

**#2** (Physical Activity):ti,ab,kw OR (Exercise):ti,ab,kw OR (Physical fit*):ti,ab,kw OR (Sport* participation):ti,ab,kw OR (Sport* activit*):ti,ab,kw

**#3** (Active lifestyle):ti,ab,kw OR (Movement behavio*):ti,ab,kw OR (MVPA):ti,ab,kw OR (Moderate to vigorous physical activity):ti,ab,kw OR (Step count*):ti,ab,kw

**#4** (Energy expenditur*):ti,ab,kw OR (Daily activit*):ti,ab,kw OR (Walk*):ti,ab,kw OR (Jog*):ti,ab,kw OR (Run):ti,ab,kw

**#5** #1 OR #2 OR #3 OR #4

**#6** MeSH descriptor: [Digital Health] explode all trees

**#7** MeSH descriptor: [Wearable Electronic Devices] explode all tree**s**

**#8** MeSH descriptor: [Mobile Applications] explode all trees

**#9** MeSH descriptor: [Telemedicine] explode all trees

**#10** (Digital health):ti,ab,kw OR (Mobile health):ti,ab,kw OR (Electronic health):ti,ab,kw OR (eHealth):ti,ab,kw OR (mHealth):ti,ab,kw

**#11** (Wearable device*):ti,ab,kw OR (Fitness track*):ti,ab,kw OR (Fitness tracking device):ti,ab,kw OR (Activity monitor*):ti,ab,kw OR (Smartphone app*):ti,ab,kw

**#12** (Mobile applicat*):ti,ab,kw OR (Mobile phone app):ti,ab,kw OR (Technology-based intervention):ti,ab,kw OR (Virtual fitness platform):ti,ab,kw OR (Online health intervention):ti,ab,kw

**#13** (Digital intervention):ti,ab,kw OR (Gamified health):ti,ab,kw OR (Health gamification):ti,ab,kw OR (Exergame):ti,ab,kw OR (Active video game):ti,ab,kw

**#14** (Internet-based intervention):ti,ab,kw OR (Text message intervention):ti,ab,kw OR (SMS-based intervention):ti,ab,kw OR (Social media intervention*):ti,ab,kw OR (Digital behavior change intervention):ti,ab,kw

**#15** #6 OR #7 OR #8 OR #9 OR #10 OR #11 OR #12 OR #13 OR #14

**#16** MeSH descriptor: [Adolescent] explode all trees

**#17** (Adolescen*):ti,ab,kw OR (Teenag*):ti,ab,kw OR (Secondary school student):ti,ab,kw OR (High school student):ti,ab,kw OR (Middle school student):ti,ab,kw

**#18** (School-age* child*):ti,ab,kw

**#19** #16 OR #17 OR #18

**#20** #5 AND #15 AND #19

**7** The final keywords chain used in the systematic literature search in **ProQuest** were as follows(Used the following Filters: English, all open to access,,article published from January 1, 2014, to June 30, 2025.):

**Initial Search Date:**2025.6.22

**Last Search Date:**2025.8.3

**ProQuest** **(via ProQuest platform ) Number of records retrieved** :**78**

**S1** title(Exercise) OR abstract(Physical Activity OR Physical fit* OR Sport* participation OR Sport* activit* OR Active lifestyle OR Movement behavio* OR MVPA OR Moderate to vigorous physical activity OR Step count* OR Energy expenditur* OR Daily activit* OR Walk* OR Jog OR Run)

**S2** title('digital health'OR 'wearable electronic devices' OR 'Mobile Applications'OR 'Telemedicine') OR abstract(Mobile health OR Electronic health OR eHealth OR mHealth OR Wearable device* OR Fitness track* OR Fitness tracking device OR Activity monitor* OR Smartphone app* OR Mobile phone app OR Technology-based intervention OR Virtual fitness platform OR Online health intervention OR Digital intervention OR Gamified health OR Health gamification OR Exergame OR Active video game OR Internet-based intervention OR Text message intervention OR SMS-based intervention OR Social media intervention* OR Digital behavior change intervention)

**S3** title(Adolescent) OR abstract(Teenag* OR Secondary school student OR High school student OR Middle school student OR School-age* child*)

**S4** [S1] AND [S2] AND [S3]

**8 Google Scholar** ( supplementary search )

**Initial Search Date :** 2025.6.22

**Last Search Date :** 2025.8.3

**Results screened ( first n = 283 ) Results were sorted by relevance ( default ).**

The following free-text search terms were used to identify relevant studies:

( Exercise OR Physical Activity OR Physical fitness OR Sports participation OR Sports activity OR ACTIVE lifestyle OR Movement behavior OR MVPA OR Moderate to vigorous physical activity OR Step COUNT OR Energy expenditure OR Daily activity OR Walking OR Jogging OR Running ) AND ( "Digital Health" OR "wearable electronic devices" OR "Mobile Applications" OR "Telemedicine" OR "Mobile health" OR "Electronic health" OR "eHealth" OR "mHealth" OR "Wearable device" OR "Fitness tracker" OR "Fitness tracking device" OR "Activity monitor" OR "Smartphone app" OR "Mobile phone app" OR "Technology-based intervention" OR "Virtual fitness platform" OR "Online health intervention" OR "Digital intervention" OR "Gamified health" OR "Health gamification" OR "Exergame" OR "Active video game" OR "Internet-based intervention" OR "Text message intervention" OR "SMS-based intervention" OR "Social media intervention" OR "Digital behavior change intervention" ) AND ( Adolescent OR Teenag* OR Secondary school student OR High school student OR Middle school student OR School-aged children )
