# Supplementary material for: Digital Health Interventions to Promote Physical Activity Among Adolescents: Systematic Review
Source: J Med Internet Res. 2026 Feb 27;28:e82395. doi: 10.2196/82395 (PMC13148130; doi:10.2196/82395)

**Multimedia Appendix 3**

# Supplementary Figures and Tables

## **1.1Supplementary Figures**


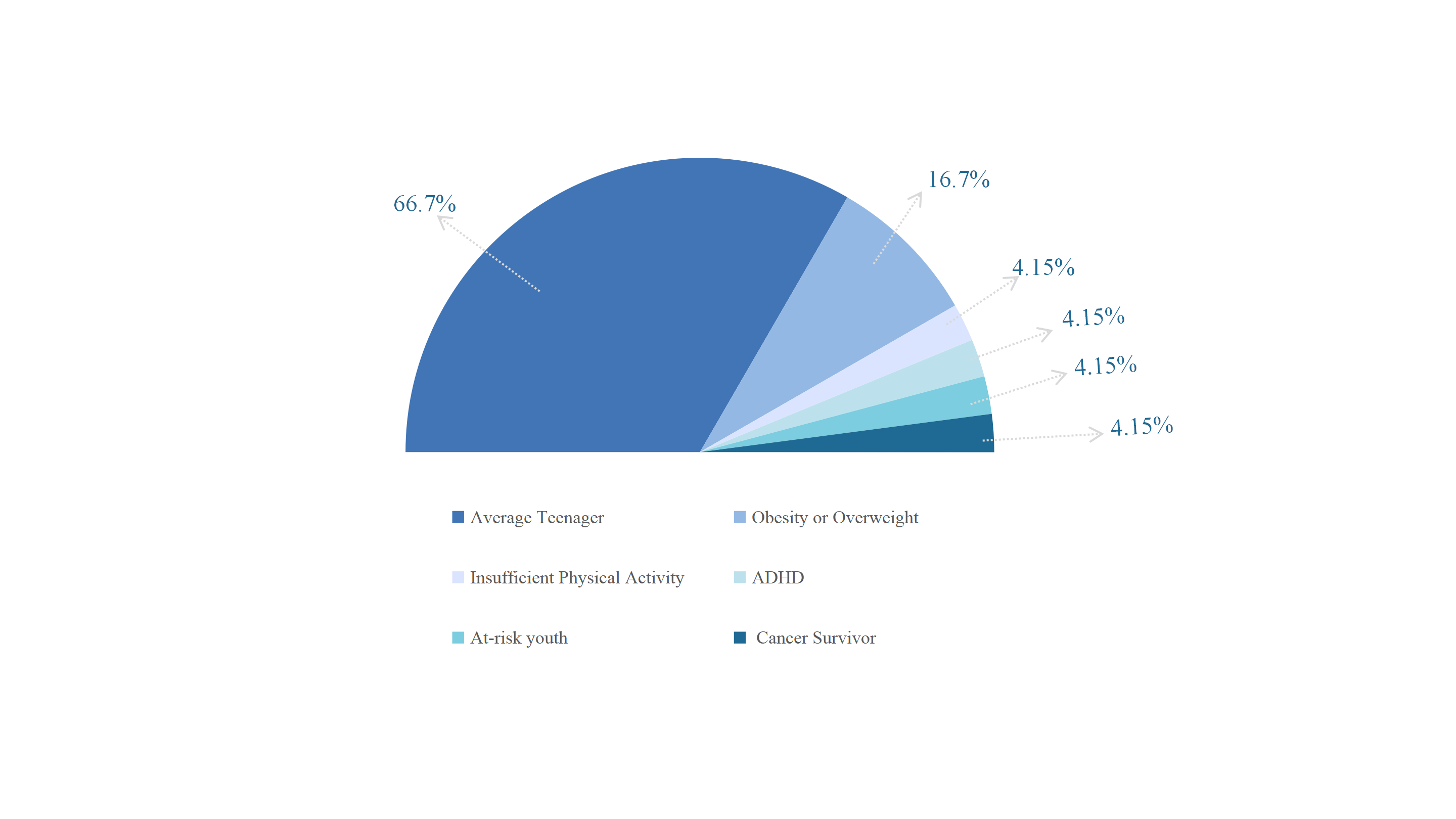


**Supplementary Figure 2. Overview of Subgroup Distribution Among Study Population (Proportional Representation)**

| 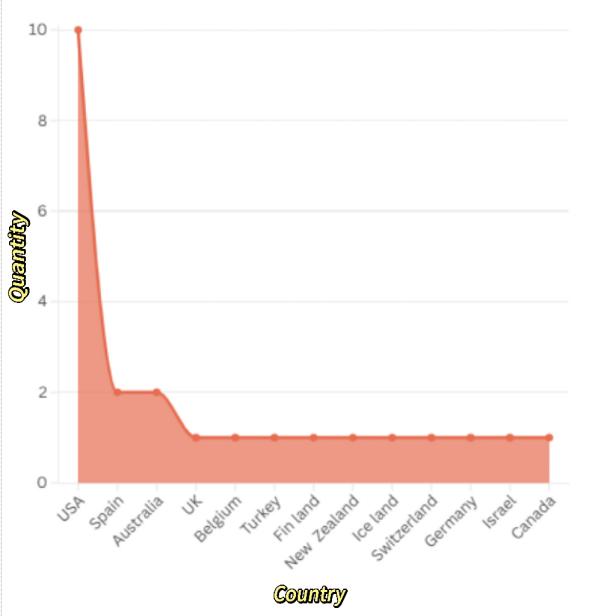 |
| --- |

**Supplementary Figure 3. Geographical Distribution of Study Samples (N=24)**

## **1.2Supplementary Tables**

**Table1 Characteristics of included studies. (N=24)(Full version)**

| Study | Age, mean (SD); grade; gender; condition; population | Effect measure(s) | Region | Study design | Sample size | Intervention duration | Digital health interventions | Effects of digital health interventions | Follow-up/sample attrition | Quality |
| --- | --- | --- | --- | --- | --- | --- | --- | --- | --- | --- |
| Soltero et al [15] | - - 14.9 (SD 0.91); 53% female; obese/overweight | Qualitative only | Spain | QR^a^ | 15 | 12 weeks | Wearable device (Fitbit Watch) + SMS text messaging | High engagement in self-monitoring behavior and perceived increase in activity | N/A | High |
| Goodyear et al [16] | - - 13-14; 53% female; general adolescents | Qualitative only | The United Kingdom | QR | 100 | 8 weeks | Wearable device (Fitbit) + virtual platform (with activity goal setting) | Encouraged youth to engage in more physical exercise | N/A | High |
| Van Dyck et al [17] | - - 12-14; 49% female; general adolescents | Qualitative only | Belgium | QR | 41 | 1 week | SMS text messaging/chatbot + social media (Facebook) + SMS | Facebook and SMS text messaging were considered promising methods for physical activity interventions among vocational school adolescents | N/A | Moderate |
| Dinç et al [18] | - - 14.27 (SD 0.44); 56% female; general adolescents | Mean difference (SE) and *P* values | Turkey | CSS^b^ | 563 | N/A | mHealth^c^: smartphone app | Effectively improves physical activity levels and health awareness | N/A | Moderate |
| Mojica et al [19] | - - 11-14; 100% female; general adolescents | Odds ratio with 95% CI; mean (SD); and significance test | The United States | CSS | 110 | N/A | Social media: cell phones, computers, game consoles, and internet | Increased physical activity and more frequent participation in daily physical education classes after the intervention | N/A | High |
| Ng et al [20] | - 11-15; 42% female; general adolescents | Odds ratio with 95% CI adjusted for covariates | Finland | CSS | 9940 | N/A | Wearable device: physical activity tracker (smartwatches and heart rate monitors) | Positive correlation between physical activity behavior and use of wearables/apps; further research needed to verify relationship | N/A | High |
| Mendoza et al [21] | - - Intervention: 16.9 (SD 1.5); 41.4% female; cancer survivors   Control: 16.3 (SD 1.5); 40% female; cancer survivors | Mean difference (minutes/day) for moderate-to-vigorous physical activity and sedentary time; and *P* values | The United States | RCT^d^ | 60 | 10 weeks | mHealth: Fitbit + Facebook | Demonstrates good feasibility and acceptability | N/A | Moderate |
| Larsen et al [22] | - - 14.7 (SD 2.1); insufficient physical activity | Mean difference in activity minutes/week; pre-post activity comparison; and baseline and follow-up SD | The United States | RCT | 21 | 12 weeks | Virtual platform: intervention website | Shows good feasibility and acceptability, with a significant increase in self-reported physical activity levels | Follow-up | High |
| Thompson et al [23] | - - 14-17; 51.88% female; general adolescents (group: control, pedometer only, pedometer + goal prompts, pedometer + goal prompts + SDT texts) | Mean differences between baseline and follow-up for each group, *P* values, and *F* values | The United States | RCT | 160 | 12 weeks | mHealth: smartphone (SMS text messaging) + pedometer | Moderate increase in average daily steps and moderate-to-vigorous physical activity | Follow-up/sample attrition | Moderate |
| Guthrie et al [24] | - - 12-14; 55% female; general adolescents | Mean difference between intervention and control groups; *P* values; and *r* | The United States | RCT | 31 | 6 weeks | Virtual platform: online intervention system (Zamzee) | Positive impact on moderate-to-vigorous physical activity levels | N/A | Moderate |
| Direito et al [25] | - - 14-17; general adolescents | Mean difference for continuous outcomes; *P* values; and CIs | New Zealand | RCT | 51 | 8 weeks | mHealth: smartphone (immersive application) | Demonstrated feasibility | N/A | Moderate |
| Chen et al [26] | - - 14.9 (SD 1.7); 42% female; obesity or overweight | *z* scores and *P* values | The United States | RCT | 40 | 6 months | mHealth: + wearable device + virtual platform: SMS text messaging, Fitbit Flex, and online educational program | Increased number of days per week engaged in physical activity | Follow-up | Moderate |
| Caillaud et al [27] | - - Intervention: 10.9 (SD 0.7); 51% female; general adolescents   Control: 10.4   - (SD 0.5); 62% female; general adolescents | Mean difference between pre- and postintervention for both intervention and control groups and *P* values | Australia | RCT | 83 | 5 weeks | Virtual platform: app (iEngage Program) | Improved physical activity goals, academic performance, and moderate-to-vigorous physical activity levels | N/A | Moderate |
| Egilsson et al [28] | - - 15.6 (SD 0.26); 41% female; general adolescents | Attrition rate; correlation (Rt); and mean difference | Iceland | RCT | 41 | 6 weeks | mHealth: smartphone (Mobile Health Program) | Feasible and usable | Sample attrition | Moderate |
| Staiano et al [29] | - - 11.2 (SD 0.8); 46% female; obesity or overweight | Mean difference between the intervention and control groups and *P* values | The United States | RCT | 46 | 24 weeks | Game: Squad Intervention (gaming console) | Improved physical activity levels | N/A | Moderate |
| Stasinaki et al [30] | - - PathMate2: 12.6 (range 11.4-16.9); 38.9% female; obesity or overweight   - Control: 13.7 (range 10.9-16.8); 46.2% female; obesity or overweight | Mean differences; *P* values (Wilcoxon signed-rank); Spearman rank; and generalized estimating equations | Switzerland | RCT | 41 | 5.5 months | mHealth: smartphone (PathMate2) | Significant and sustained improvements in physical capacity and body composition | Sample attrition | Moderate |
| Ortega  and Cushing  [31] | - - 13-18; 45% female; general adolescents | Regression coefficients (*B*); 95% CIs; and *P* values | The United States | MMS^e^ | 10 | 27 days | SMS text messaging/chatbot: text bot (TAGS) | Increased participation, with observable changes in physical activity before and after the intervention | N/A | Moderate |
| Willinger et al [32] | - - 12.6 (SD 1.7);27% female; general adolescents | Mean differences and *P* values | Germany | MMS | 10 | 4 months | mHealth: smartphone (KIJANI app) | Helps oneself and others to be more active in daily life | N/A | Moderate |
| Schoenfelder et al [33] | - - 15.5 (SD 1.4); 54% female; with attention-deficit/hyperactivity disorder | Mean differences; *P* values; and 95% CIs | The United States | MMS | 11 | 4 weeks | mHealth + Fitbit Flex + Facebook | Average weekly step count increased; intervention shows promise for promoting physical activity in adolescents with attention-deficit/hyperactivity disorder | N/A | Low |
| Koorts et al [34] | - - 13.7 (SD 0.4); 49%female; general adolescents | Mean differences; *P* values; percentage; and qualitative data | Australia | MMS | 142 | 12 weeks | mHealth + wearable device (wrist-worn Fitbit) | Perceived short-term positive effect on exercise motivation | Follow-up (teacher) | Moderate |
| Glaser et al [35] | - - Ninth to eleventh grade; 28% female; at-risk youth | *P* values and adjusted *R*^2^ | Israel | QES^f^ | 180 | 8 months | Virtual platform: Friends Online Intervention Program—Zoom/video chat with online physical activities and dialogue | The program effectively increased physical activity among adolescents and reduced risky behaviors | N/A | Moderate |
| Garde et al [36] | - - 8-13; general adolescents | Mean differences; *P* values; 95% CIs | Canada | QES | 47 | 2 weeks | mHealth: smartphone game (Mobile Kids Monster Manor) | After the intervention, more physical activity was observed; the game’s role in promoting activity needs further validation | N/A | Moderate |
| Mateo-Orcajada et al [37] | - - 13.96 (SD 1.21); 48% female; general adolescents | Mean differences; *P* values; 95% CIs; *F* values | Spain | QES | 400 | 10 weeks | mHealth: mobile app | Postintervention improvements in physical activity level, body composition, and physical fitness quality | Sample attrition | High |
| Cushing et al [38] | - - 13-18; 75% female; general adolescents | Mean differences and β | The United States | QES | 40 | 3 weeks | SMS/chatbot: tailored SMS text messaging intervention | Increased physical activity and reduced sedentary behavior | N/A | Moderate |

^a^QR: quantitative research.

^b^CSS: cross-sectional study.

^c^mHealth: mobile health.

^d^RCT: randomized controlled trial.

^e^MMS: mixed methods study.

^f^QES: quasi-experimental study.

**Table3. Comparison of Features and Effectiveness Across**

**Three Types of Digital Health Interventions**

| Type | Single-Driver Intervention | Multi modal Integrated Intervention | Interaction-Enhanced Intervention |
| --- | --- | --- | --- |
| Technological Approach | Primarily m Health (smartphone-based) | m Health + wearable devices + virtual platforms | Gamification mechanisms + social interaction |
| Stage | Initial stage of behavior change | Stage targeting medium- to long-term health management | Stage of behavior maintenance and reinforcement |
| Method | Encourage individuals to take action: set goals, send reminders, record activities | Build a closed-loop system: behavior tracking, real-time feedback, goal adjustment | Stimulate adolescents’ emotional and social motivation; enhance engagement: competitions, likes, virtual rewards, socializing, feedback, gamified mechanisms |
| Strengths | 1. Emphasizes convenience and functionality of technology tools 2. Helps build basic behavioral habits | 1. Offers layered design in data collection, feedback, and behavior guidance 2. Stronger data connectivity enables dynamic monitoring and adjustment by researchers | 1. Focuses on motivation and emotional engagement 2. Improves adherence and enthusiasm for participation |
| Limitations | 1. Limited by user motivation, tech literacy, and feedback mechanisms 2. Behavior change may be hard to sustain over time | 1. High system complexity and operational burden 2. Strong dependence on devices, high implementation costs 3. Adherence may be difficult to maintain 4. Delays in data integration and feedback | Effectiveness may vary among individuals with low social motivation or limited digital access |

## References

1. Soltero EG, Musaad SM, O’Connor TM, Thompson D, Norris K, Beech BM. Feasibility of Fit24, a Digital Diabetes Prevention Program for Hispanic Adolescents: Qualitative Evaluation Study. JMIR Form Res 2024 May 17;8:e54595. doi: [10.2196/54595](https://doi.org/10.2196/54595).PMID:38758584.
2. Goodyear VA, Kerner C, Quennerstedt M. Young people’s uses of wearable healthy lifestyle technologies; surveillance, self-surveillance and resistance. Sport, Education and Society 2019 Mar 24;24(3):212–225. doi: [10.1080/13573322.2017.1375907](https://doi.org/10.1080/13573322.2017.1375907)
3. Van Dyck D, D’Haese S, Plaete J, De Bourdeaudhuij I, Deforche B, Cardon G. Opinions towards physical activity interventions using Facebook or text messaging: Focus group interviews with vocational school‐aged adolescents. Health Soc Care Community 2019 May;27(3):654–664. doi: [10.1111/hsc.12679](https://doi.org/10.1111/hsc.12679).PMID:30398291.
4. Dinç F, Kurt A, Yıldız D. The use of mobile health applications in the development of a healthy lifestyle of adolescents: A cross-sectional study. J Pediatr Nurs 2025 July;83:1–6. doi: [10.1016/j.pedn.2025.04.012](https://doi.org/10.1016/j.pedn.2025.04.012).PMID:40273677.
5. Mojica CM, Parra-Medina D, Yin Z, Akopian D, Esparza LA. Assessing Media Access and Use Among Latina Adolescents to Inform Development of a Physical Activity Promotion Intervention Incorporating Text Messaging. Health Promotion Practice 2014 July;15(4):548–555. doi: [10.1177/1524839913514441](https://doi.org/10.1177/1524839913514441)
6. Ng K, Kokko S, Tammelin T, Kallio J, Belton S, O’Brien W, Murphy M, Powell C, Woods C. Clusters of Adolescent Physical Activity Tracker Patterns and Their Associations With Physical Activity Behaviors in Finland and Ireland: Cross-Sectional Study. J Med Internet Res 2020 Sept 1;22(9):e18509. doi: [10.2196/18509](https://doi.org/10.2196/18509).PMID:24357863.
7. Mendoza JA, Baker KS, Moreno MA, Whitlock K, Abbey‐Lambertz M, Waite A, Colburn T, Chow EJ. A Fitbit and Facebook mHealth intervention for promoting physical activity among adolescent and young adult childhood cancer survivors: A pilot study. Pediatr Blood Cancer 2017 Dec;64(12). doi: [10.1002/pbc.26660](https://doi.org/10.1002/pbc.26660).PMID:28618158.
8. Larsen B, Benitez T, Cano M, Dunsiger SS, Marcus BH, Mendoza-Vasconez A, Sallis JF, Zive M. Web-Based Physical Activity Intervention for Latina Adolescents: Feasibility, Acceptability, and Potential Efficacy of the Niñas Saludables Study. J Med Internet Res 2018 May 9;20(5):e170. doi: [10.2196/jmir.9206](https://doi.org/10.2196/jmir.9206).PMID:29743151.
9. Thompson D, Cantu D, Ramirez B, Cullen KW, Baranowski T, Mendoza J, Anderson B, Jago R, Rodgers W, Liu Y. Texting to Increase Adolescent Physical Activity: Feasibility Assessment. Am J Health Behav 2016 July 1;40(4):472–483. doi: [10.5993/AJHB.40.4.9](https://doi.org/10.5993/AJHB.40.4.9).PMID:27338994.
10. Guthrie N, Bradlyn A, Thompson SK, Yen S, Haritatos J, Dillon F, Cole SW. Development of an Accelerometer-Linked Online Intervention System to Promote Physical Activity in Adolescents. PLOS One 2015;10(5):e0128639. doi: [10.1371/journal.pone.0128639](https://doi.org/10.1371/journal.pone.0128639).PMID:26010359.
11. Direito A, Jiang Y, Whittaker R, Maddison R. Smartphone apps to improve fitness and increase physical activity among young people: protocol of the Apps for IMproving FITness (AIMFIT) randomized controlled trial. BMC Public Health 2015 Dec;15(1):635. doi: [10.1186/s12889-015-1968-y](https://doi.org/10.1186/s12889-015-1968-y).PMID:26159834.
12. Chen J-L, Guedes CM, Cooper BA, Lung AE. Short-Term Efficacy of an Innovative Mobile Phone Technology-Based Intervention for Weight Management for Overweight and Obese Adolescents: Pilot Study. Interact J Med Res 2017 Aug 2;6(2):e12. doi: [10.2196/ijmr.7860](https://doi.org/10.2196/ijmr.7860).PMID:28768612.
13. Caillaud C, Ledger S, Diaz C, Clerc G, Galy O, Yacef K. iEngage: A digital health education program designed to enhance physical activity in young adolescents. PLOS One 2022;17(10):e0274644. doi: [10.1371/journal.pone.0274644](https://doi.org/10.1371/journal.pone.0274644).PMID:36197890.
14. Egilsson E, Bjarnason R, Njardvik U. Usage and Weekly Attrition in a Smartphone-Based Health Behavior Intervention for Adolescents: Pilot Randomized Controlled Trial. JMIR Form Res 2021 Feb 17;5(2):e21432. doi: [10.2196/21432](https://doi.org/10.2196/21432).PMID:33481750.
15. Staiano AE, Beyl RA, Guan W, Hendrick CA, Hsia DS, Newton RL. Home‐based exergaming among children with overweight and obesity: a randomized clinical trial. Pediatric Obesity 2018 Nov;13(11):724–733. doi: [10.1111/ijpo.12438](https://doi.org/10.1111/ijpo.12438).PMID:30027607.
16. Stasinaki A, Büchter D, Shih C-HI, Heldt K, Güsewell S, Brogle B, Farpour-Lambert N, Kowatsch T, l’Allemand D. Effects of a novel mobile health intervention compared to a multi-component behaviour changing program on body mass index, physical capacities and stress parameters in adolescents with obesity: a randomized controlled trial. BMC Pediatr 2021 Dec;21(1):308. doi: [10.1186/s12887-021-02781-2](https://doi.org/10.1186/s12887-021-02781-2).PMID:34243738.
17. Ortega A, Cushing CC. Design of a Temporally Augmented Text Messaging Bot to Improve Adolescents’ Physical Activity and Engagement: Proof-of-Concept Study. JMIR Form Res 2024 Oct 10;8:e60171. doi: [10.2196/60171](https://doi.org/10.2196/60171).PMID:39388222.
18. Willinger L, Schweizer F, Böhm B, Scheller DA, Jonas S, Oberhoffer-Fritz R, Müller J, Reimer LM. Evaluation of the gamified application KIJANI to promote physical activity in children and adolescents: A multimethod study. DIGITAL HEALTH 2024 Jan;10:20552076241271861. doi: [10.1177/20552076241271861](https://doi.org/10.1177/20552076241271861).PMID:39161345.
19. Schoenfelder E, Moreno M, Wilner M, Whitlock KB, Mendoza JA. Piloting a mobile health intervention to increase physical activity for adolescents with ADHD. Preventive Medicine Reports 2017 June;6:210–213. doi: [10.1016/j.pmedr.2017.03.003](https://doi.org/10.1016/j.pmedr.2017.03.003).PMID:28373931.
20. Koorts H, Salmon J, Timperio A, Ball K, Macfarlane S, Lai SK, Brown H, Chappel SE, Lewis M, Ridgers ND. Translatability of a Wearable Technology Intervention to Increase Adolescent Physical Activity: Mixed Methods Implementation Evaluation. J Med Internet Res 2020 Aug 7;22(8):e13573. doi: [10.2196/13573](https://doi.org/10.2196/13573).PMID:32763872.
21. Glaser M, Green G, Barak S, Bord S, Levi S, Jakobovich R, Dunsky A, Zigdon A, Zwilling M, Tesler R. The effects of the Friendship Online Intervention Program on physical activity, substance abuse, psychosomatic symptoms, and well‐being among at‐risk youth. J Adolesc 2024 Feb;96(2):251–265. doi: [10.1002/jad.12272](https://doi.org/10.1002/jad.12272).PMID:37985148.
22. Garde A, Umedaly A, Abulnaga SM, Robertson L, Junker A, Chanoine JP, Ansermino JM, Dumont GA. Assessment of a Mobile Game (“MobileKids Monster Manor”) to Promote Physical Activity Among Children. Games for Health Journal 2015 Apr;4(2):149–158. doi: [10.1089/g4h.2014.0095](https://doi.org/10.1089/g4h.2014.0095).PMID: 26181809.
23. Mateo-Orcajada A, Abenza-Cano L, Albaladejo-Saura MD, Vaquero-Cristóbal R. Mandatory after-school use of step tracker apps improves physical activity, body composition and fitness of adolescents. Educ Inf Technol 2023 Aug;28(8):10235–10266. doi: [10.1007/s10639-023-11584-0](https://doi.org/10.1007/s10639-023-11584-0).PMID:36714445.
24. Cushing CC, Bejarano CM, Ortega A, Sayre N, Fedele DA, Smyth JM. Adaptive mHealth Intervention for Adolescent Physical Activity Promotion. J Pediatr Psychol 2021 June 3;46(5):536–546. doi: [10.1093/jpepsy/jsaa125](https://doi.org/10.1093/jpepsy/jsaa125).PMID:33484137.


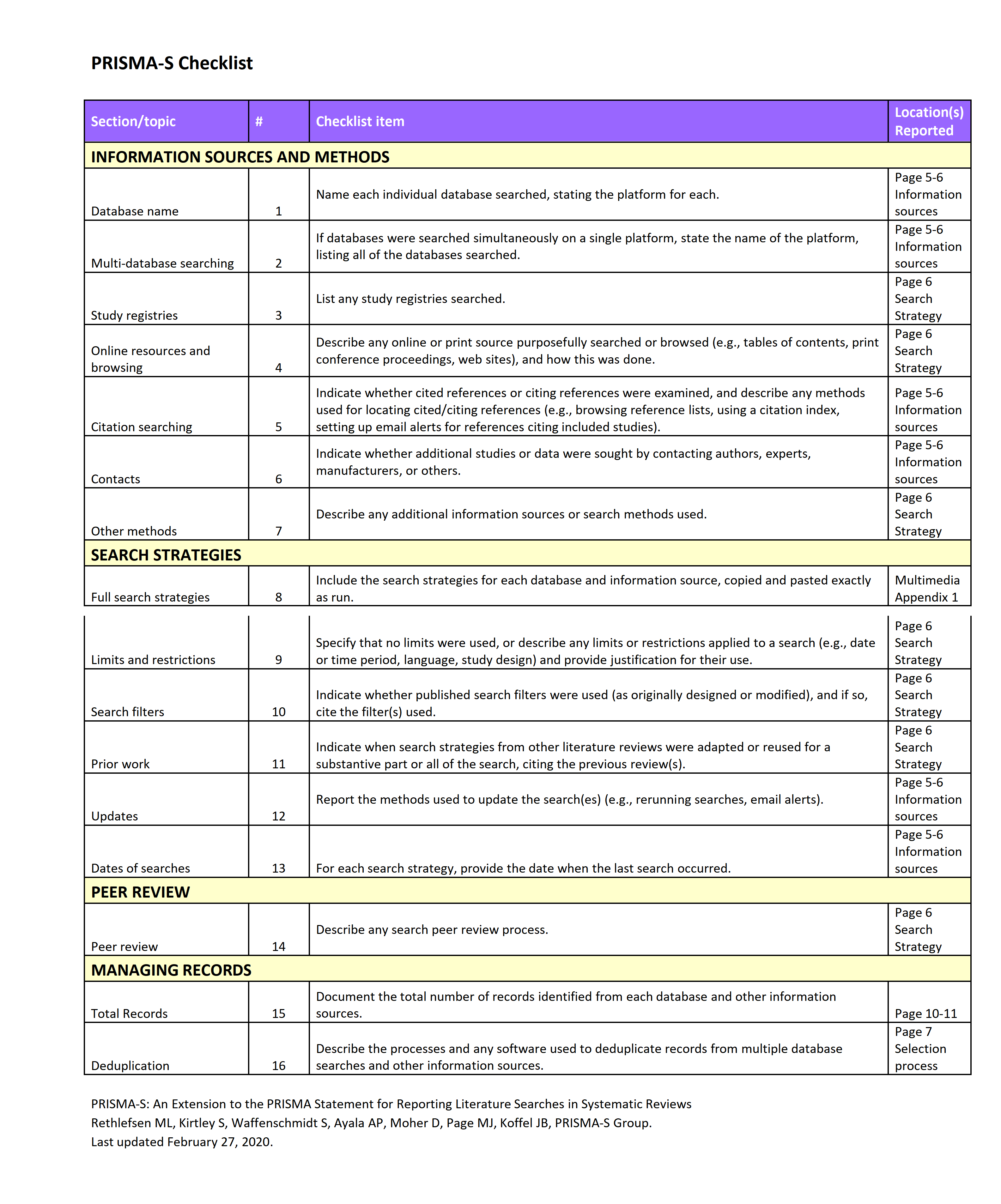

Supplement: Multimedia Appendix 3 [file jmir_v28i1e82395_app3.docx]
